# Supplementary material for: Decomposing response inhibition: A POMDP model
Source: PLoS Comput Biol. 2026 Sep 25;22(9):e1014068. doi: 10.1371/journal.pcbi.1014068 (PMC13693991; doi:10.1371/journal.pcbi.1014068)
Supplement: Supplementary file 1 [file pcbi.1014068.s001.pdf]

S1 Appendix: Supplementary Methods and Results

Contents

A Mathematical details of the POMDP model **2**

A.1 Derivation of stop process inference 2

A.2 Formulation for independent stop signal task 3

A.3 Transition probabilities 3

A.4 Tensor-based value iteration 4

A.5 Full probabilistic policy visualization 4

A.6 Stop prior sensitivity analysis 9

B TeSBI architecture and validation **10**

B.1 TeSBI architecture 10

B.2 Transformer encoded representations 12

C POMDP model selection and validation **14**

C.1 Model specification 15

C.2 Model selection 15

C.3 Parameter recovery analysis 16

C.4 Posterior predictive checks 16

D Comparison with alternative models **17**

E Computational attributes **19**

E.1 Aggregate distributions of computational attributes 19

E.2 Parameter regression and interaction analysis 19

E.3 Predictor sets comparison 21

F Model-agnostic statistical analysis **21**

A Mathematical details of the POMDP model

A.1 Derivation of stop process inference

This section details the recursive Bayesian updates for the intermediate stop-signal belief $\zeta_t^\nu := P(\theta^\nu \leq t \mid y_{1:t}^\nu)$ and the stop-trial belief $\sigma_t^\nu := P(s^\nu = 1 \mid y_{1:t}^\nu)$.

1. Adjusted hazard rate (h_t^ν) First, we derive the adjusted hazard rate, which represents the probability that the stop signal occurs at time t, given it has not occurred previously. This term must account for the uncertainty regarding the trial type s^ν.

$$\begin{aligned} h_t^\nu &:= P(\theta^\nu = t \mid \theta^\nu > t-1) \\ &= \frac{P(\theta^\nu = t, s^\nu = 1)}{P(\theta^\nu > t-1)} \\ &= \frac{P(\theta^\nu = t \mid s^\nu = 1)P(s^\nu = 1)}{P(\theta^\nu > t-1 \mid s^\nu = 1)P(s^\nu = 1) + P(\theta^\nu > t-1 \mid s^\nu = 0)P(s^\nu = 0)} \end{aligned} \tag{S1}$$

Using the geometric prior $P(\theta^\nu = t|s^\nu = 1) = \lambda(1-\lambda)^{t-1}$ and noting that for go trials ($s^\nu = 0$), the stop signal never occurs (equivalent to $\theta^\nu \to \infty$, thus $P(\theta^\nu > t-1|s^\nu = 0) = 1$), we obtain:

$$h_t^\nu = \frac{\lambda(1-\lambda)^{t-1} \cdot \tilde{r}^\nu}{(1-\lambda)^{t-1} \cdot \tilde{r}^\nu + (1-\tilde{r}^\nu)}. \tag{S2}$$

2. Stop signal belief prediction ($\tilde{\zeta}_t^\nu$) Before observing y_t^ν, the agent computes the *predicted* belief that the signal has occurred by time t. This combines the previous posterior with the probability of a new onset:

$$\begin{aligned} \tilde{\zeta}_t^\nu &:= P(\theta^\nu \leq t \mid y_{1:t-1}^\nu) \\ &= P(\theta^\nu \leq t-1 \mid y_{1:t-1}^\nu) + P(\theta^\nu = t \mid \theta^\nu > t-1, y_{1:t-1}^\nu)P(\theta^\nu > t-1 \mid y_{1:t-1}^\nu) \\ &= \zeta_{t-1}^\nu + h_t^\nu \cdot (1-\zeta_{t-1}^\nu). \end{aligned} \tag{S3}$$

Complementarily, the probability that the signal has *not* occurred by time t is:

$$1 - \tilde{\zeta}_t^\nu = (1-\zeta_{t-1}^\nu)(1-h_t^\nu). \tag{S4}$$

3. Stop signal belief correction (ζ_t^ν) Upon observing y_t^ν, we update the belief via Bayes' rule.

$$\begin{aligned} \zeta_t^\nu &= P(z_t^\nu = 1 \mid y_{1:t}^\nu) \\ &= \frac{P(y_t^\nu \mid z_t^\nu = 1)P(z_t^\nu = 1 \mid y_{1:t-1}^\nu)}{P(y_t^\nu \mid y_{1:t-1}^\nu)} \end{aligned} \tag{S5}$$

Expanding the denominator using the law of total probability and substituting Eq. S4:

$$P(y_t^\nu \mid y_{1:t-1}^\nu) = P(y_t^\nu \mid z_t^\nu = 1)\tilde{\zeta}_t^\nu + P(y_t^\nu \mid z_t^\nu = 0)(1-\tilde{\zeta}_t^\nu). \tag{S6}$$

This yields the update equation used in the main text.

4. Stop trial belief (σ_t^ν) Finally, the probability that the current trial is a stop trial is the sum of two disjoint possibilities: (a) the signal has already occurred, or (b) the signal has not occurred yet but the trial is essentially a stop trial (signal will occur in the future).

$$\begin{aligned} \sigma_t^\nu &:= P(s^\nu = 1 \mid y_{1:t}^\nu) \\ &= \underbrace{P(s^\nu = 1 \mid \theta^\nu \leq t, y_{1:t}^\nu)}_{=1} \underbrace{P(\theta^\nu \leq t \mid y_{1:t}^\nu)}_{\zeta_t^\nu} + P(s^\nu = 1 \mid \theta^\nu > t, y_{1:t}^\nu) \underbrace{P(\theta^\nu > t \mid y_{1:t}^\nu)}_{1-\zeta_t^\nu} \\ &= \zeta_t^\nu + (1-\zeta_t^\nu) \cdot \underbrace{P(s^\nu = 1 \mid \theta^\nu > t)}_{\text{Residual Probability}} \end{aligned} \tag{S7}$$

The residual probability represents the likelihood that a trial is a stop trial given no signal has appeared by time t:

$$P(s^\nu = 1 \mid \theta^\nu > t) = \frac{P(\theta^\nu > t \mid s^\nu = 1)P(s^\nu = 1)}{P(\theta^\nu > t)} = \frac{(1-\lambda)^t \tilde{r}^\nu}{(1-\lambda)^t \tilde{r}^\nu + (1-\tilde{r}^\nu)}. \tag{S8}$$

Combining these terms yields the final expression for σ_t^ν.

A.2 Formulation for independent stop signal task

In standard SST designs (e.g., auditory stop signals), the stop signal does not visually mask or interrupt the go cue. Consequently, the two sensory streams are effectively independent. Our framework accommodates this by removing the conditional dependency of the go observation on the stop signal state.

1. Observation function for go process Unlike the main model where the stop signal ($z_t^\nu = 1$) alters the go observation process, here we assume $P(x_t^\nu \mid d^\nu, z_t^\nu) = P(x_t^\nu \mid d^\nu)$. The go cue remains visible and unmasked throughout the trial. Hence, the observation probabilities are time-invariant and independent of stop signal onset (Table A).

Table A. Observation likelihoods for go process (independent SST)

**State** d^ν	$P(x_t^\nu = L \mid d^\nu)$	$P(x_t^\nu = R \mid d^\nu)$	$P(x_t^\nu = N \mid d^\nu)$
0	$(1-\chi')\chi$	$(1-\chi')(1-\chi)$	χ'
1	$(1-\chi')(1-\chi)$	$(1-\chi')\chi$	χ'

2. Decoupled inference and transitions Under this independence assumption, the belief update for the go process no longer requires marginalization over the stop signal belief ζ_t^ν. The update simplifies to:

$$\beta_t^\nu = \frac{\beta_{t-1}^\nu P(x_t^\nu \mid d^\nu = 1)}{\beta_{t-1}^\nu P(x_t^\nu \mid d^\nu = 1) + (1-\beta_{t-1}^\nu) P(x_t^\nu \mid d^\nu = 0)}. \tag{S9}$$

Consequently, the joint transition probability factorizes into two independent components:

$$P(x_{t+1}, y_{t+1} \mid \mathbf{b}_t) = P(x_{t+1} \mid \beta_t) \cdot P(y_{t+1} \mid \zeta_t), \tag{S10}$$

where $P(x_{t+1} \mid \beta_t)$ depends solely on the go belief, and $P(y_{t+1} \mid \zeta_t)$ depends solely on the stop process dynamics. This formulation recovers the standard assumption of parallel, independent processing channels used in race models.

A.3 Transition probabilities

To evaluate the action-value of Wait (W), the model must predict future observations by marginalizing over latent states. The transition probabilities $P(x_{t+1}, y_{t+1} \mid \mathbf{b}_t, W)$ are derived by marginalizing over the future stop signal state z_{t+1}:

$$P(x_{t+1}, y_{t+1} \mid \mathbf{b}_t, W) = \sum_{z_{t+1} \in \{0,1\}} P(x_{t+1} \mid \beta_t, z_{t+1}) \cdot P(y_{t+1} \mid z_{t+1}) \cdot P(z_{t+1} \mid \zeta_t) \tag{S11}$$

where the transition probability for the go observation x_{t+1} marginalizes over the latent true direction d:

$$P(x_{t+1} \mid \beta_t, z_{t+1}) = \beta_t \cdot P(x_{t+1} \mid d = 1, z_{t+1}) + (1-\beta_t) \cdot P(x_{t+1} \mid d = 0, z_{t+1}) \tag{S12}$$

Given the approximation that the stop signal is functionally persistent once it appears, the transition probabilities for the stop signal state are given by the predicted belief $\tilde{\zeta}_{t+1}$:

$$P(z_{t+1} = 1 \mid \zeta_t) = \tilde{\zeta}_{t+1} = \zeta_t + (1 - \zeta_t)h_{t+1} \tag{S13}$$

$$P(z_{t+1} = 0 \mid \zeta_t) = 1 - \tilde{\zeta}_{t+1} \tag{S14}$$

The primitive observation likelihoods $P(x \mid d, z)$ and $P(y \mid z)$ correspond to the functions defined in Tables 2 and 3 of the main text.

A.4 Tensor-based value iteration

To enable scalable inference over the ABCD cohort, we vectorized the POMDP value iteration using tensor operations over a discretized belief space. This replaces slow, sequential loop-based computations with highly optimized matrix operations.

1. Discretized belief grid We discretize the continuous belief state space $(\beta, \zeta) \in [0, 1]^2$ into a uniform grid of resolution $N_\beta \times N_\zeta$ (in our implementation, $N_\beta = N_\zeta = 80$).

$$\mathbf{B} = \{(\beta_i, \zeta_j) \mid i \in 1 \dots N_\beta, j \in 1 \dots N_\zeta\}$$

At any time step t, the value function V_t and action-value functions $Q_t(\cdot, a)$ are represented as matrices of shape $N_\beta \times N_\zeta$.

2. Transition tensor construction The core computational bottleneck is the expectation over future observations. We precompute a transition tensor $\mathcal{T}_t$ that encodes the probability of transitioning to any future observation pair (x', y') given any current belief state on the grid:

$$\mathcal{T}_t[i, j, x', y'] = P(x', y' \mid \beta_i, \zeta_j) \in \mathbb{R}^{N_\beta \times N_\zeta \times |\mathcal{X}| \times |\mathcal{Y}|}$$

where $|\mathcal{X}| = 3$ (Left, Right, Null) and $|\mathcal{Y}| = 3$ (Absence, Presence, Null). This tensor is computed via the factorized likelihoods described in Eq. S11, efficiently broadcasting the independent updates for the Go process (dependent on β) and Stop process (dependent on ζ).

3. Vectorized Bellman update The expected value of waiting, $Q_t(\mathbf{b}, W)$, involves an integral over future beliefs. In the discretized space, this becomes a tensor contraction. Let $(\beta_i, \zeta_j, x', y')$ be the deterministic next-step belief indices given current state (i, j) and observation (x', y'). We compute the next-step value tensor of shape $N_\beta \times N_\zeta \times |\mathcal{X}| \times |\mathcal{Y}|$ by gathering values from V_{t+1} according to the updated belief indices. The Q-value tensor for the Wait action is then computed in a single operation:

$$Q_t(\cdot, \cdot, W) = \sum_{x' \in \mathcal{X}} \sum_{y' \in \mathcal{Y}} \mathcal{T}_t[\cdot, \cdot, x', y'] \odot \mathcal{V}_{t+1}[\cdot, \cdot, x', y'] \tag{S15}$$

where $\odot$ denotes element-wise multiplication. This vectorization allows us to solve the full dynamic programming problem for a single participant in milliseconds.

A.5 Full probabilistic policy visualization

This section provides a comprehensive overview of the probabilistic policy and belief state evolution across all 40 time steps, categorized by the five possible trial outcomes.

The background heatmaps illustrate the softmax policy across the entire joint belief space, comprising the go belief (β_t^ν) and stop signal belief (ζ_t^ν), mapping regions that favor Wait (light gray), Go-Left (yellow), or Go-Right (blue). Heatmap colors are gamma-corrected for visual clarity (see Optimal control

in the Results section for details). As the deadline approaches, the go regions progressively expand inward, reflecting the model's increasing urgency to act to avoid a timeout.

Overlaid scatter points trace the simulated belief trajectories from $6,000$ go trials (right go cue) and $1,200$ stop trials (right go cue, SSD=11), with dot size representing the density of belief states. Categorizing these trajectories by outcome reveals the distinct patterns of observation accumulation driving the model's behavior. Rapid vertical shifts lead to early responses in Go Success and Go Error trials; weak directional observations leave states stranded in the Wait region during Go Missing trials; and rapid rightward shifts upon Stop signal detection successfully suppress responses in Stop Success trials, provided the initial Go observations did not accumulate fast enough to cause a Stop Error.

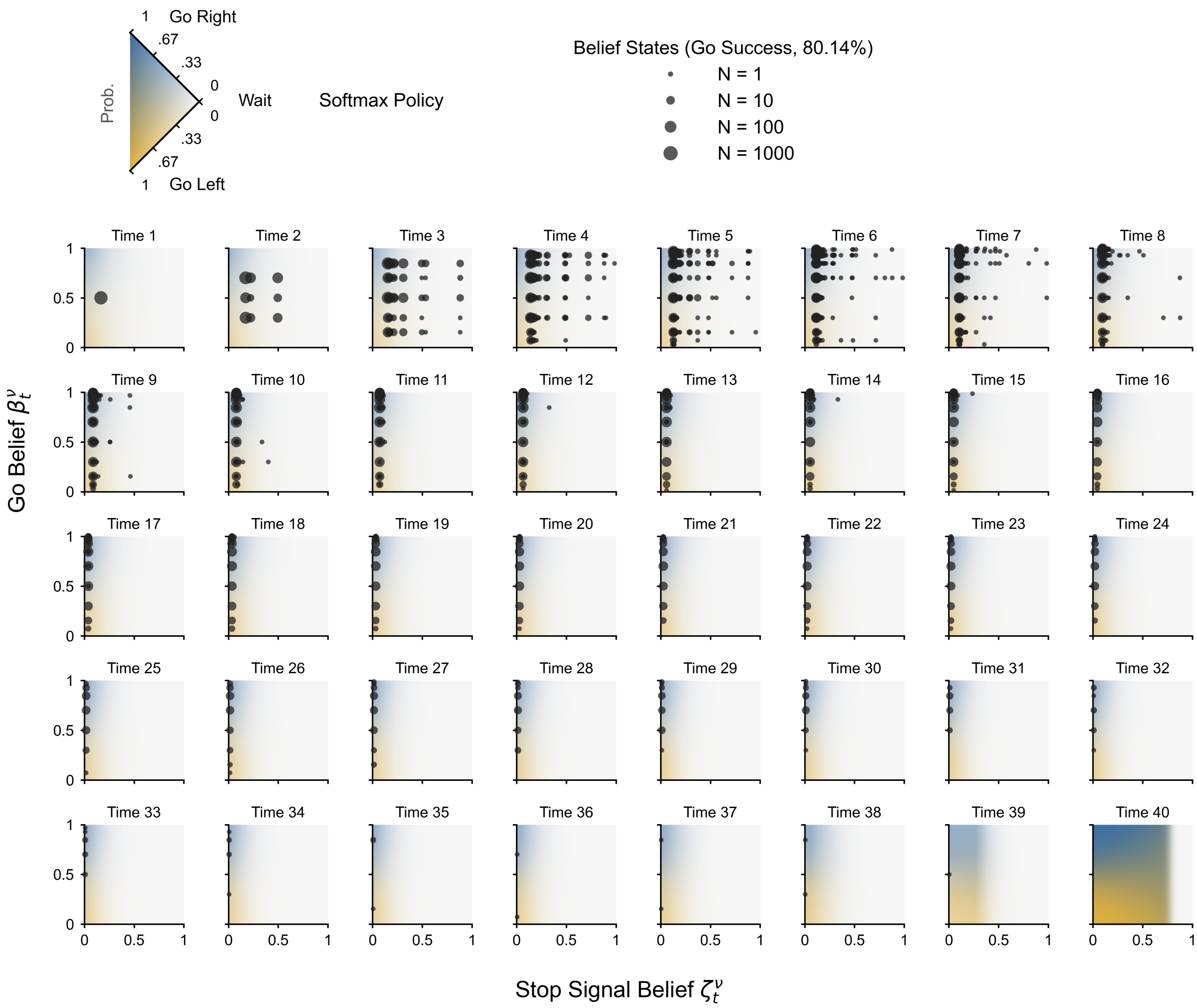

Fig A. Softmax policy and belief states for Go Success trials. Background heatmaps represent action probabilities from the softmax policy. Scatter points show simulated trajectories from $6,000$ go trials (right go cue).

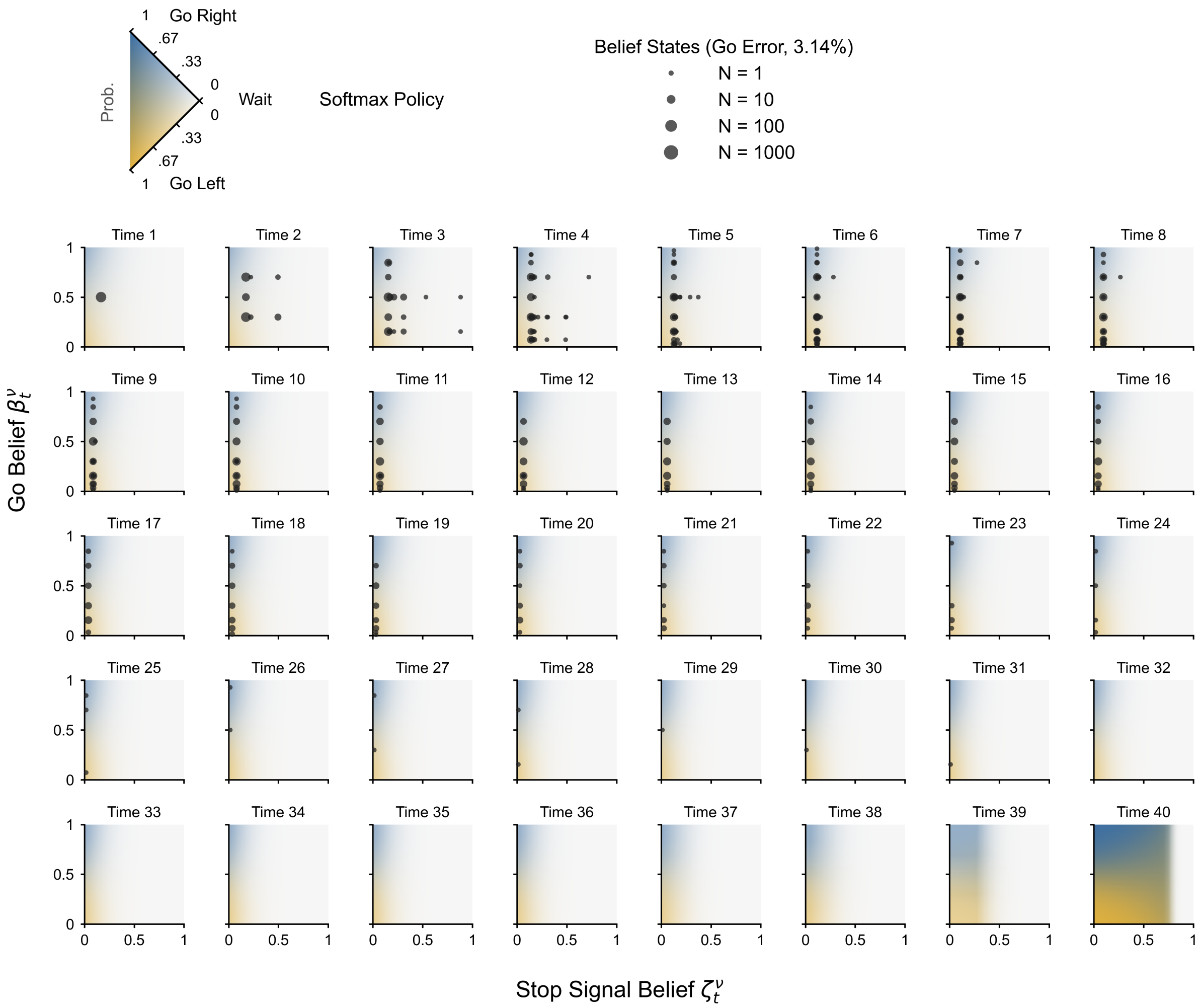

Fig B. Softmax policy and belief states for Go Error trials. Background heatmaps represent action probabilities from the softmax policy. Scatter points show simulated trajectories from 6,000 go trials (right go cue).

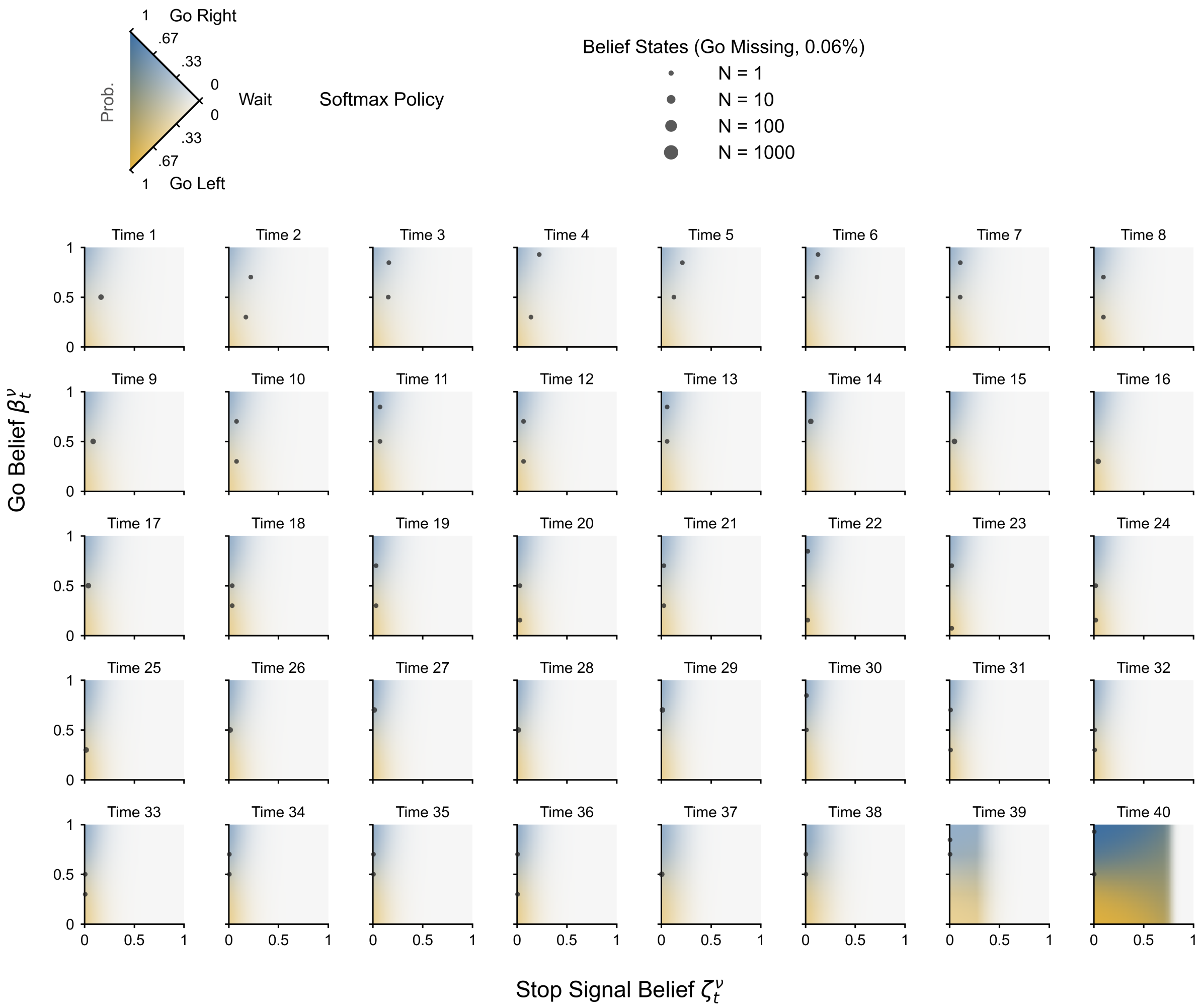

Fig C. Softmax policy and belief states for Go Missing trials. Background heatmaps represent action probabilities from the softmax policy. Scatter points show simulated trajectories from $6,000$ go trials (right go cue).

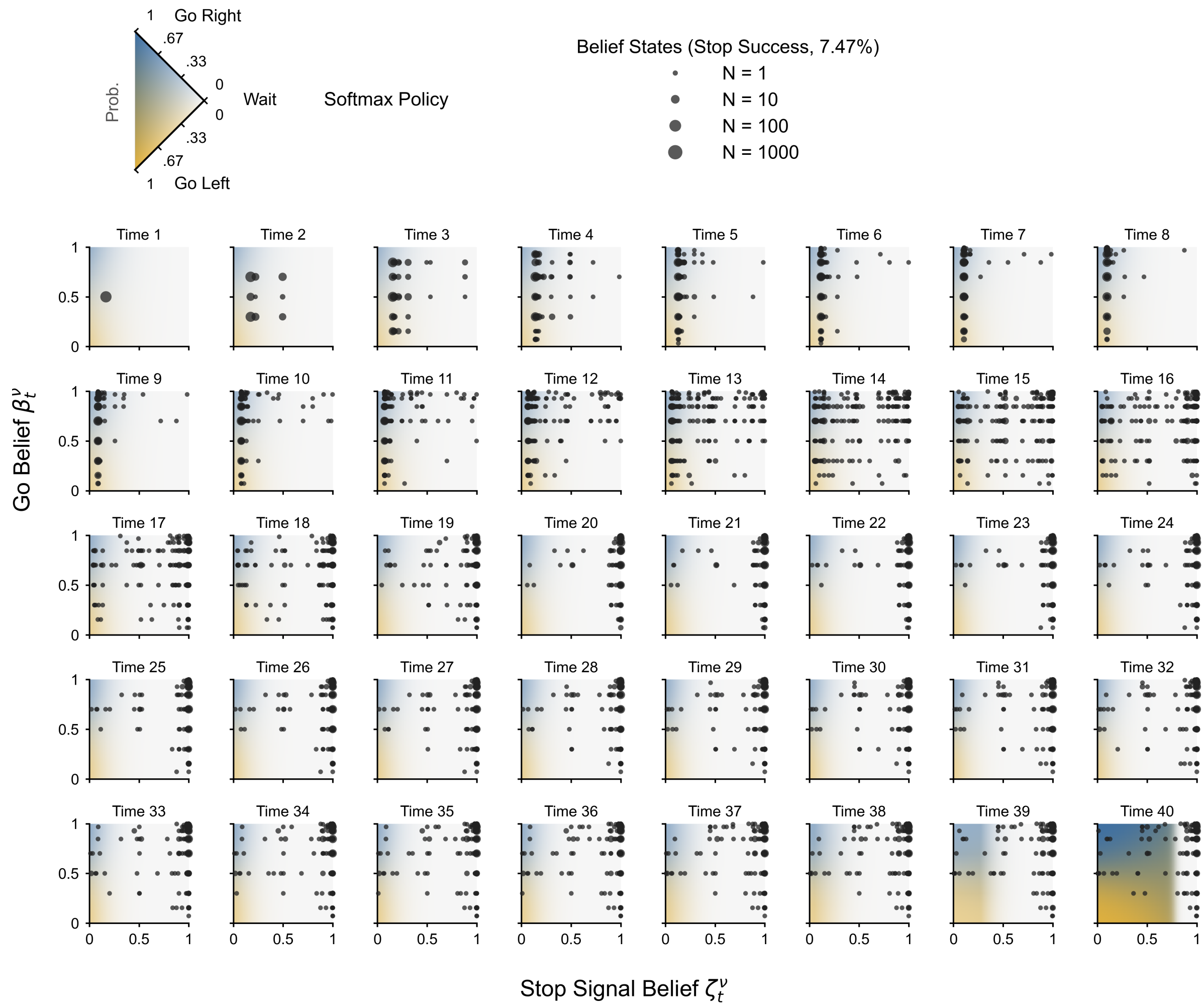

Fig D. Softmax policy and belief states for Stop Success trials. Background heatmaps represent action probabilities from the softmax policy. Scatter points show simulated trajectories from $1,200$ stop trials (right go cue, SSD=11).

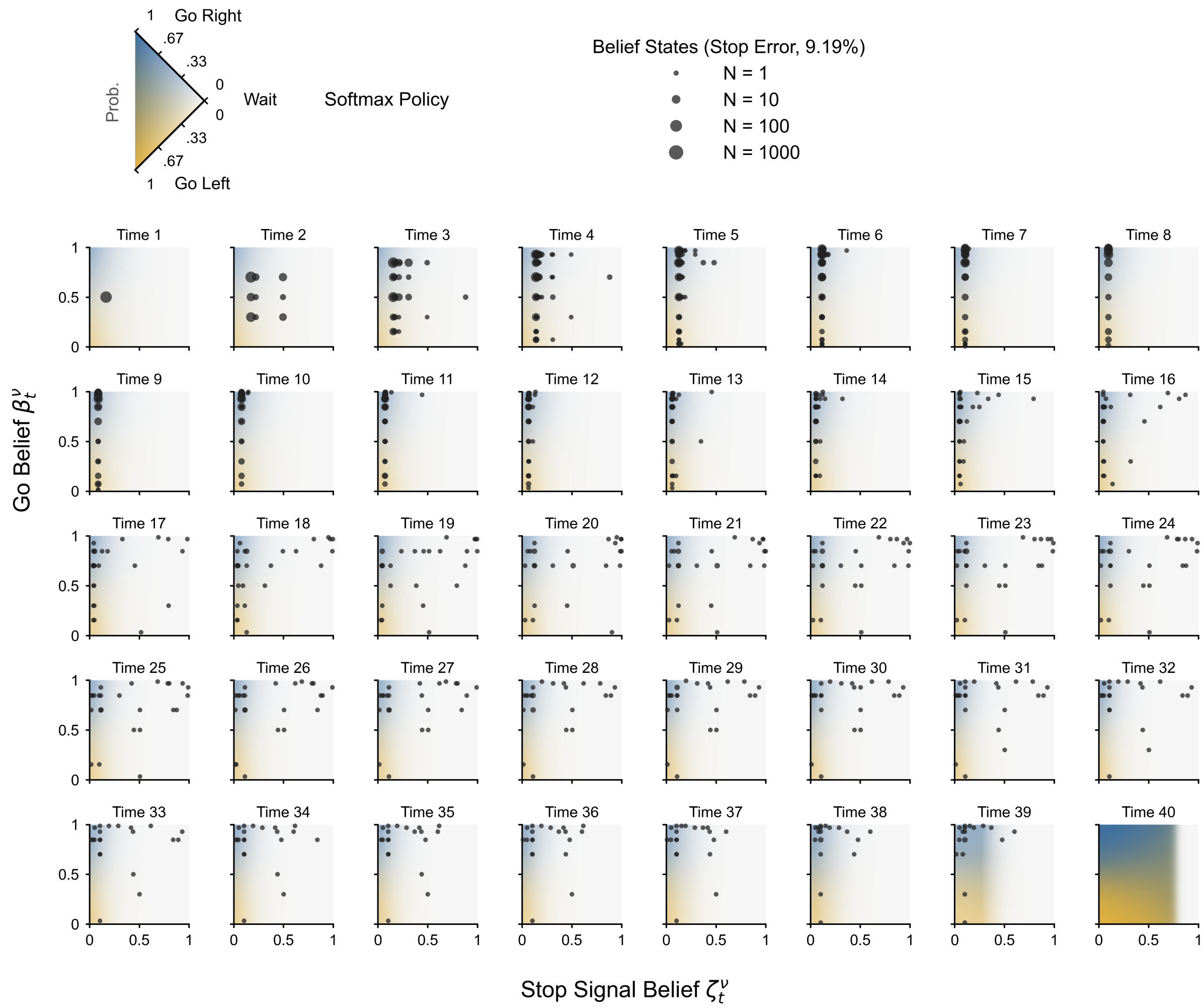

Fig E. Softmax policy and belief states for Stop Error trials. Background heatmaps represent action probabilities from the softmax policy. Scatter points show simulated trajectories from $1,200$ stop trials (right go cue, SSD=11).

A.6 Stop prior sensitivity analysis

In the ABCD task, stop trials account for only 1/6 of the total trials (60/360), which limits the natural fluctuation of participants' across-trial stop prior prediction ($\tilde{r}^\nu$). As shown in Fig F, simulating the POMDP model across a realistic range of prior probabilities (0.05 to 0.30) generates small but observable shifts in performance, such as slight increases in Go reaction times, as well as in Stop trial accuracy and error reaction times. Given this restricted range, we fixed $\tilde{r}^\nu$ to 1/6 for the current study to simplify the model and focus on estimating within-trial dynamics. This step allows us to establish stable within-trial parameters first, which can then serve as a baseline for future work to free the stop prior and investigate across-trial learning effects.

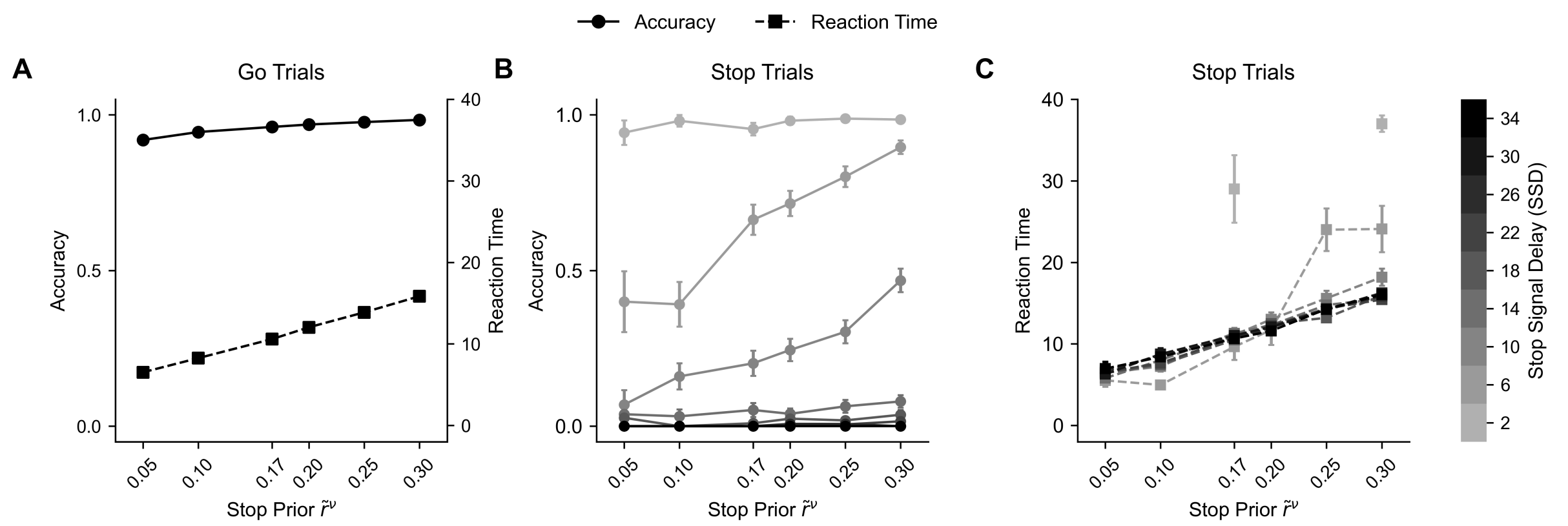

Fig F. Sensitivity of POMDP behavioral metrics to stop trial priors. Simulated outputs across varying predicted stop prior ($\tilde{r}^\nu$), centered around the ABCD SST baseline stop rate (1/6). (A) Go accuracy (solid/circles; left axis) and reaction times (dashed/squares; right axis). (B-C) Stop accuracy and reaction times across Stop Signal Delays (SSD: 2–34, grayscale). Error bars denote standard error of the mean (s.e.m.).

B TeSBI architecture and validation

B.1 TeSBI architecture

The TeSBI (Transformer-encoded Simulation-Based Inference) pipeline employs an end-to-end architecture, jointly optimizing a Transformer encoder and a Neural Spline Flow density estimator to perform amortized inference of subject-level parameter posteriors.

The overall network architecture is illustrated in Fig G. The procedure for both the amortized training phase and the posterior inference phase is detailed in Algorithm 1.

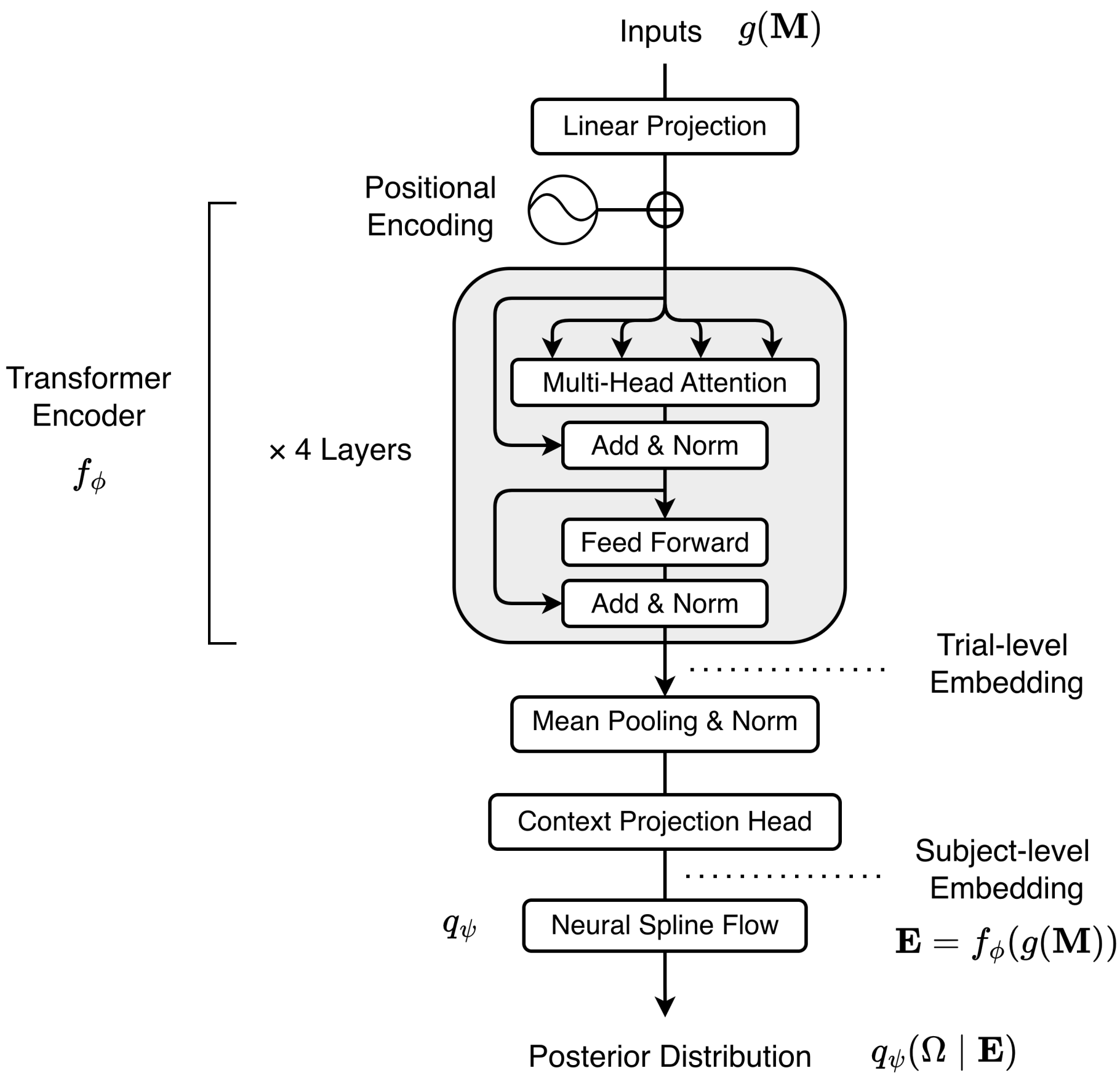

Fig G. TeSBI architecture. The input feature sequence $g(\mathbf{M})$ is linearly projected and processed by a 4-layer Transformer encoder f_ϕ to extract trial-level embeddings. These embeddings are aggregated via global mean pooling and refined by a context projection head to yield the subject-level summary embedding $\mathbf{E}$. Finally, this embedding directly conditions a Neural Spline Flow density estimator to approximate the subject-level posterior distribution $q_\psi(\Omega \mid \mathbf{E})$ of the POMDP parameters.

Algorithm 1 Transformer-encoded Simulation-Based Inference (TeSBI)

Notation: Ω: POMDP parameters; $\mathbf{M}$: trial-by-trial behavioral measurement; $g(\cdot)$: feature engineering operator; $f_\phi(\cdot)$: Transformer encoder with weights ϕ; $\mathbf{E} = f_\phi\big(g(\mathbf{M})\big)$: context embedding; $q_\psi(\Omega \mid \mathbf{E})$: Neural Spline Flow density estimator with weights ψ.

Stage 1: Amortized Training

Initialize network parameters ϕ and ψ.
Generate offline dataset:
for $j = 1, \ldots, N_{\text{sim}}$ **do**
  Sample parameters $\Omega^{(j)} \sim p(\Omega)$
  Generate synthetic behavioral sequence $\mathbf{M}^{(j)} \leftarrow \text{POMDP}(\Omega^{(j)})$
  Store parameter-sequence tuple $(\Omega^{(j)}, \mathbf{M}^{(j)})$
end for
Joint Optimization:
repeat
  Sample a mini-batch of tuples $\{(\Omega^{(b)}, \mathbf{M}^{(b)})\}_{b=1}^{B}$
  Extract engineered features $g(\mathbf{M}^{(b)})$
  Compute context embeddings $\mathbf{E}^{(b)} = f_\phi(g(\mathbf{M}^{(b)}))$
  Compute Negative Log-Likelihood: $\mathcal{L}(\phi, \psi) = -\frac{1}{B}\sum_{b=1}^{B} \log q_\psi\big(\Omega^{(b)} \mid \mathbf{E}^{(b)}\big)$
  Update ϕ and ψ jointly using gradient descent on $\mathcal{L}(\phi, \psi)$
until validation loss converges (early stopping)
Output: Trained amortized estimator $q_\psi(\Omega \mid f_\phi(\cdot))$.

Stage 2: Posterior Inference

for each participant u with empirically observed sequence $\mathbf{M}_{\text{obs}}^{(u)}$ **do**
  Compute subject-specific embedding $\mathbf{E}_{\text{obs}}^{(u)} = f_\phi\big(g(\mathbf{M}_{\text{obs}}^{(u)})\big)$ via the Transformer encoder
  Compute the subject-specific posterior distribution $q_\psi\big(\Omega \mid \mathbf{E}_{\text{obs}}^{(u)}\big)$ via the NSF estimator
  Draw K samples $\Omega_k^{(u)}$ from this posterior and extract the mean $\hat{\Omega}^{(u)}$ for subsequent analyses
end for

Implementation details The end-to-end TeSBI pipeline was implemented in PyTorch, utilizing the `zuko` library (v1.6.0) for the normalizing flow estimation.

The 18-dimensional trial-level features were first linearly projected into a continuous latent space. The Transformer encoder then operated on this projected model dimension of 128 with sinusoidal positional encodings. It comprised 4 layers with 8 attention heads each, and a feedforward dimension of 512. Trial-level representations were aggregated via global mean pooling over the temporal dimension, followed by a context projection head (comprising layer normalization, a linear projection, ReLU activation, and a final linear layer) to yield a 128-dimensional summary embedding. For density estimation, we employed a Neural Spline Flow conditioned directly on this embedding, utilizing 2 hidden layers with 256 features each.

Amortized training was performed on a dataset of $N_{\text{sim}} = 100{,}000$ simulated behavioral sessions, partitioned into an 80% training set and a 20% validation set. The unified network jointly minimized the negative log-likelihood of the ground-truth parameters given the behavioral sequences. Optimization was conducted using the AdamW optimizer (batch size 128, initial learning rate 10^{-3}, weight decay 10^{-5}). To ensure stable optimization across the joint architecture, gradients were clipped to a maximum norm of 5.0. Furthermore, we employed a learning rate scheduler (reducing the learning rate by a factor of 0.5 upon a validation loss plateau of 5 epochs) and an early stopping mechanism (with a patience of 15 epochs) to prevent overfitting.

B.2 Transformer encoded representations

Our Transformer encoder compresses the sequential data from 360 trials into a 128-dimensional embedding vector, $\mathbf{E} = f_\phi\big(g(\mathbf{M})\big)$. This approach maps the complex, dynamic behavioral profile of each

participant onto a single point in a high-dimensional latent space ($\mathbb{R}^{128}$). Fig H shows the Pearson correlation coefficients between model-agnostic behavioral metrics and the latent embeddings, showing some strong relationships.

To examine the validity of the learned representations, we perform a Canonical Correlation Analysis (CCA) between the 128-dimensional latent embeddings and 15 extracted model-agnostic behavioral metrics. After visualizing the initial pairwise correlations in Fig HA, a permutation test (1000 iterations) reveals that all 15 canonical variates are statistically significant (all $p < 0.001$, with canonical correlations $\rho_c = 0.995, 0.983, 0.928, 0.868, 0.693, 0.625, 0.527, 0.415, 0.335, 0.287, 0.263, 0.209, 0.192, 0.179$, and 0.169). As shown in Figs HB and HC, the first canonical variate captures a substantial correlation ($\rho_c = 0.99$) and reveals how both static and dynamical behaviors are holistically encoded across the latent space, confirming the strong expressive capacity of the TeSBI embeddings.

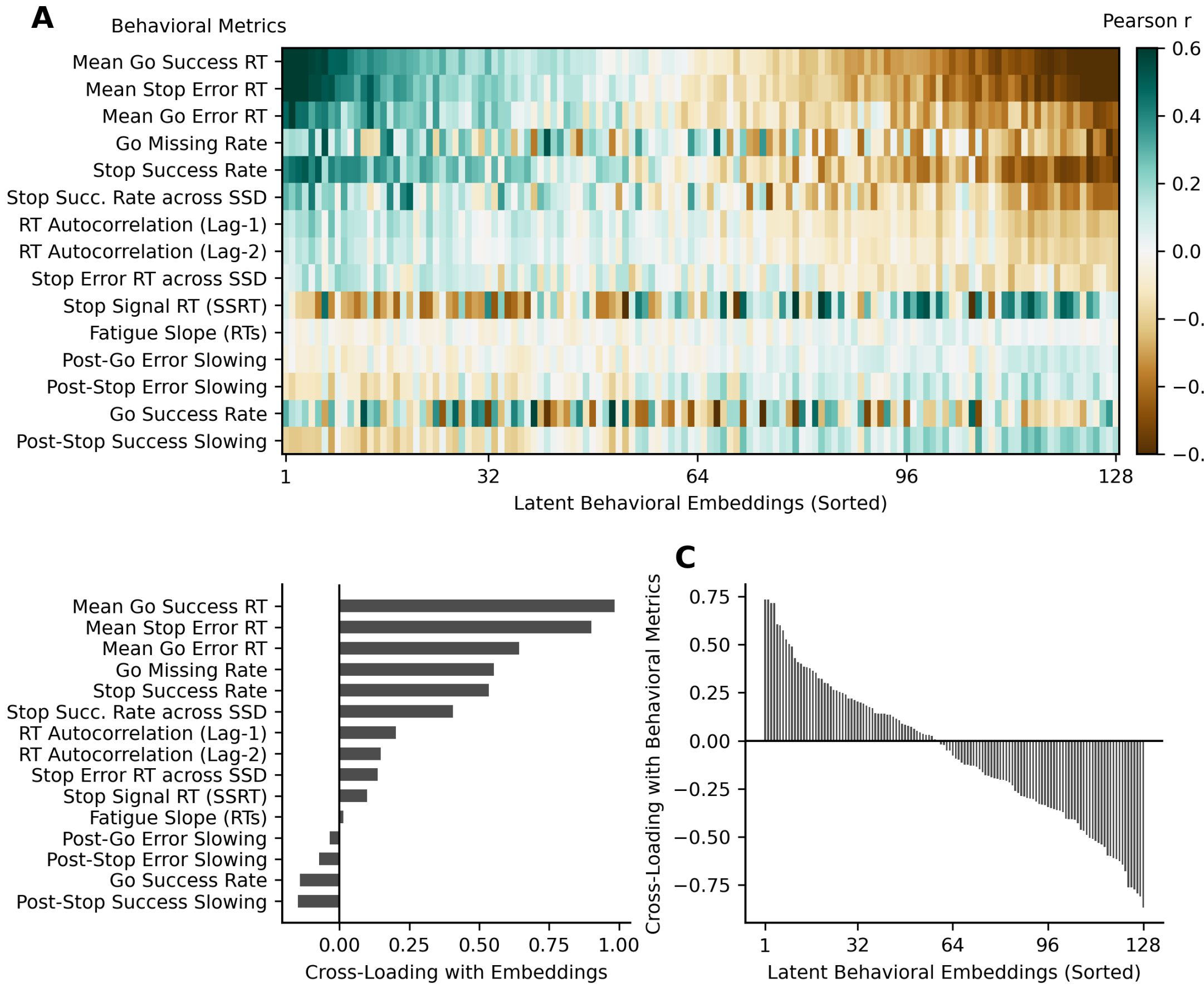

Fig H. Canonical Correlation Analysis linking learned embeddings and behavioral metrics. (A) Heatmap illustrating the pairwise Pearson correlation coefficients between the 128 latent embeddings (x-axis, sorted by canonical loadings) and the 15 extracted behavioral metrics (y-axis). (B) Sorted cross-loadings of the behavioral metrics on the first canonical variate ($\rho_c = 0.995$). The encoder effectively captures both static task performance (e.g., mean Go RT, SSRT) and temporal dynamics (e.g., post-stop slowing, RT autocorrelation). (C) Sorted cross-loadings of the latent embeddings on the first canonical variate. The graded distribution indicates that behavioral information is encoded holistically across the latent space rather than being localized to a few dominant dimensions. Only the first canonical variate that capturing the largest shared variance is visualized in panels B and C to avoid visual clutter.

To visualize the structure of these behavioral fingerprints, we apply Principal Component Analysis (PCA) to project the latent embeddings into 2D space ($\mathbb{R}^2$), capturing 39.8% of the total variance (PC 1: 24.7%, PC 2: 17.0%). As shown in Fig I, the distribution of participant embeddings forms a continuous manifold without discrete boundaries. Critically, when mapping clinical scores onto the 2D space, we observe that participants with higher ADHD scores are dispersed throughout the manifold rather than forming a distinct or isolated "disorder cluster".

This dispersion provides visual confirmation of our regression results: higher ADHD scores are not linked to a single, unique computational failure factor. Instead, higher ADHD scores are compatible with diverse computational profiles, ranging from varied sensory processing capacities to differing cost valuations. This heterogeneity supports dimensional frameworks (e.g., RDoC) that conceptualize clinical labels as broad, overlapping distributions along a continuous cognitive spectrum, rather than categorical deficits defined by a single phenotype.

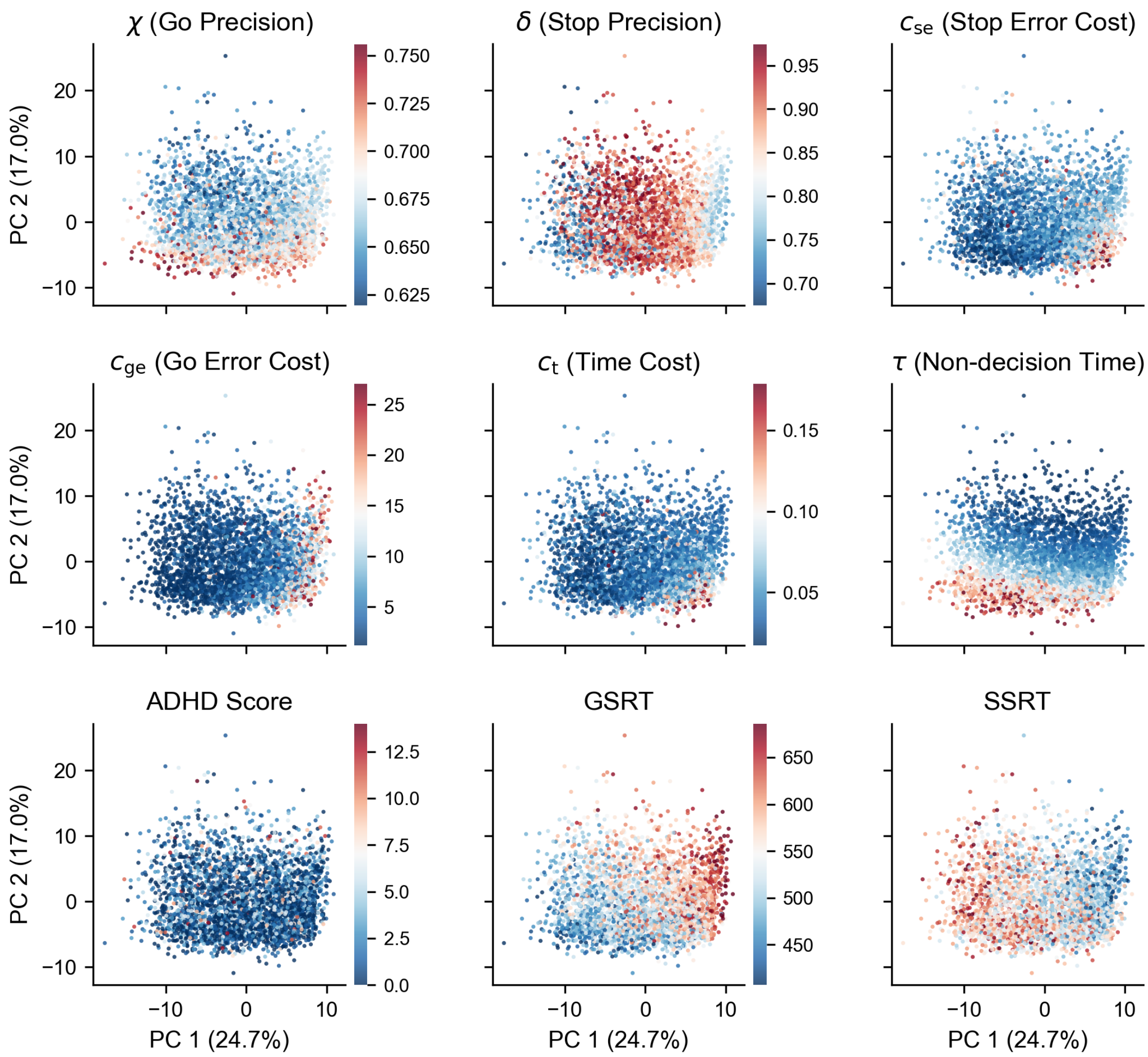

Fig I. 2D PCA projection of the behavioral embedding space. Participant embeddings are projected onto a 2D space using PCA (explaining 41.7% of total variance). Each point represents a single participant ($N = 3,567$). Panels are colored by POMDP parameters and behavioral metrics to visualize the continuous nature of the latent behavioral landscape. Notably, participants with higher ADHD scores are heterogeneously distributed throughout the space rather than clustering in a specific region. This illustrates that clinical characteristics map onto a diverse spectrum of computational attributes rather than a single isolated deficit.

C POMDP model selection and validation

To ensure parameter identifiability while preserving predictive power, we apply an iterative step-down procedure across 15 POMDP variants (Table B). Then, starting from the 10-parameter full model (M10.1), we iteratively reduce unidentifiable parameters. At each step, the parameters with the lowest recovery correlations are fixed to specific constants based on posterior quantiles (20%, 50%, or 80%).

C.1 Model specification

Table B outlines the specifications and parameter constraints across all variants. The full model (M10.1) includes ten parameters: go null (χ'), go precision (χ), stop null (δ'), stop precision (δ), go error cost (c_{ge}), go missing cost (c_{gm}), stop error cost (c_{se}), time cost (c_t), inverse temperature (φ), and non-decision time (τ).

The *Prior Range* row defines the estimation boundaries for each parameter. In the subsequent model rows, checkmarks (✓) denote free parameters estimated within these prior ranges, whereas single numerical values indicate that the parameter is fixed to a specific constant.

Table B. POMDP model specification and parameter priors

Model	χ'	χ	δ'	δ	c_{ge}	c_{gm}	c_{se}	c_t	φ	τ	$\tilde{r}^{\nu}$
**Prior Range**	$[0, 1]$	$[0.5, 1]$	$[0, 1]$	$[0.5, 1]$	$[1, 50]$	$[1, 50]$	$[1, 50]$	$[0.01, 0.5]$	$[10, 100]$	$[4, 16]$	Fixed
**M10.1**	✓	✓	✓	✓	✓	✓	✓	✓	✓	✓	**1/6**
**M8.1**	✓	✓	0.1348	✓	✓	✓	✓	✓	44.6534	✓	**1/6**
**M8.2**	✓	✓	**0.0787**	✓	✓	✓	✓	✓	**44.6534**	✓	**1/6**
**M8.3**	✓	✓	0.2441	✓	✓	✓	✓	✓	44.6534	✓	**1/6**
**M8.4**	✓	✓	0.1348	✓	✓	✓	✓	✓	37.9558	✓	**1/6**
**M8.5**	✓	✓	0.1348	✓	✓	✓	✓	✓	51.8192	✓	**1/6**
**M7.1**	✓	✓	**0.0787**	✓	✓	9.5955	✓	✓	**44.6534**	✓	**1/6**
**M7.2**	✓	✓	**0.0787**	✓	✓	13.1139	✓	✓	**44.6534**	✓	**1/6**
**M7.3**	✓	✓	**0.0787**	✓	✓	**15.7821**	✓	✓	**44.6534**	✓	**1/6**
**M7.4**	0.1026	✓	**0.0787**	✓	✓	✓	✓	✓	**44.6534**	✓	**1/6**
**M7.5**	0.2361	✓	**0.0787**	✓	✓	✓	✓	✓	**44.6534**	✓	**1/6**
**M7.6**	0.5328	✓	**0.0787**	✓	✓	✓	✓	✓	**44.6534**	✓	**1/6**
**M6.1**	**0.0798**	✓	**0.0787**	✓	✓	**15.7821**	✓	✓	**44.6534**	✓	**1/6**
**M6.2**	0.1671	✓	**0.0787**	✓	✓	**15.7821**	✓	✓	**44.6534**	✓	**1/6**
**M6.3**	0.4295	✓	**0.0787**	✓	✓	**15.7821**	✓	✓	**44.6534**	✓	**1/6**

C.2 Model selection

In order to find a model of the behavior which fits the data well, but does not overfit, we employ a step-down procedure to remove degrees of freedom that are not recoverable. In the first step of this procedure, the inverse temperature (φ) and stop null (δ') are the primary bottlenecks in M10.1, showing the worst recovery ($r = 0.30$ and $r = 0.34$, respectively; Table C). Fixing these two parameters based on the posterior quantiles of M10.1 yields five 8-parameter variants (M8.1–M8.5). We select M8.2 ($\varphi = 44.6534$, $\delta' = 0.0787$) for the next step due to its superior in-sample (Table D) and out-of-sample (Table E) posterior predictive checks.

In the second step, we address the least recoverable parameters in M8.2 by creating six 7-parameter variants. We fix either the go missing cost (c_{gm}) (M7.1–M7.3) or the go null parameter (χ') (M7.4–M7.6) to the posterior quantiles of M8.2. From these, M7.3 ($c_{\text{gm}} = 15.7821$) emerges as the best variant.

In the final step, because χ' remains the sole bottleneck in M7.3 (r = 0.6681), we further reduce the architecture to 6-parameter variants (M6.1–M6.3) by fixing χ' to the posterior quantiles of M7.3. All M6.1, M6.2 and M6.3 successfully pass the parameter recovery test for all remaining free parameters (Pearson's $r > 0.65$). Ultimately, we select M6.1 ($c_{\text{gm}} = 2.2687$) as the primary computational model. It achieves complete parameter identifiability while maintaining strong in-sample and out-of-sample predictive power (total distances of 1.88611 and 3.77383).

C.3 Parameter recovery analysis

To evaluate parameter identifiability, we simulate behavior using parameters sampled from the priors, fit the models to these simulated datasets, and calculate the Pearson correlation coefficient (r) between the ground-truth parameters and the recovered estimates.

To ensure robustness, we apply a criterion requiring $r > 0.65$ for all estimated parameters (indicated by checkmarks in the Valid column). As Table C shows, while increasing the number of free parameters enhances model flexibility to fit the data, it also introduces severe parameter unidentifiability. Our step-down procedure effectively resolves this issue. In the final selected model (M6.1), all six free parameters strictly satisfy this identifiability criterion: go precision ($r = 0.89$), stop precision ($r = 0.72$), non-decision time ($r = 0.83$), stop error cost ($r = 0.81$), time cost ($r = 0.82$), and go error cost ($r = 0.72$).

Table C. Parameter recovery correlations across model variants

**Model**	χ	δ	τ	c_{se}	c_t	c_{ge}	c_{gm}	χ'	δ'	φ	**Valid**
M10.1	0.7338	0.52576	0.76639	0.71686	0.77255	0.67475	0.25241	0.68363	0.28484	0.23538	
M8.1	0.78803	0.67084	0.76253	0.72547	0.64495	0.65898	0.19468	0.56529	—	—	
M8.2	0.75792	0.63352	0.75234	0.72376	0.68564	0.6542	0.18572	0.58766	—	—	
M8.3	0.78363	0.65966	0.75261	0.74255	0.69466	0.65715	0.25048	0.5983	—	—	
M8.4	0.77669	0.67832	0.78664	0.7536	0.66689	0.65421	0.20505	0.58863	—	—	
M8.5	0.76724	0.62577	0.7137	0.73714	0.67105	0.63331	0.26696	0.58201	—	—	
M7.1	0.77864	0.73923	0.78226	0.8094	0.74747	0.56983	—	0.66981	—	—	
M7.2	0.74035	0.74885	0.80225	0.82987	0.76128	0.60295	—	0.66004	—	—	
M7.3	0.77628	0.75358	0.78879	0.82022	0.7603	0.6681	—	0.57688	—	—	
M7.4	0.78577	0.68973	0.69869	0.81331	0.73715	0.59321	0.24727	—	—	—	
M7.5	0.80723	0.70444	0.71446	0.85211	0.78155	0.61792	0.29937	—	—	—	
M7.6	0.78866	0.70419	0.70713	0.79195	0.76916	0.57304	0.19798	—	—	—	
**M6.1**	**0.89382**	**0.72778**	**0.83096**	**0.81072**	**0.82478**	**0.72995**	—	—	—	—	✓
M6.2	0.88438	0.69043	0.84585	0.80845	0.80418	0.71801	—	—	—	—	✓
M6.3	0.89813	0.70072	0.81195	0.76931	0.76785	0.70143	—	—	—	—	✓

C.4 Posterior predictive checks

To validate the predictive power of the models, we perform in-sample posterior predictive checks (PPCs) by simulating the entire task behavior for each participant using their posterior mean estimates. We then quantify the empirical distance between the simulated and observed data across multiple metrics: choice proportions (percentages of Go Success, Go Error, Go Missing, and Stop Success) and reaction time distributions (measured via Wasserstein and Kolmogorov-Smirnov distances for Go Success and Stop Error trials).

To evaluate generalization and prevent overfitting, we further apply an out-of-sample temporal cross-validation PPC. For each participant, we use the first 80% of trials to estimate the posterior parameters and simulate the subsequent behavior. We then compute the empirical distances strictly on the withheld 20% of trials, across the same metrics.

The empirical distances for the in-sample and out-of-sample checks are summarized in Table D and Table E, respectively.

Table D. In-sample empirical distances from posterior predictive checks

	Choice Proportions				WS Dist. (RT)			KS Dist. (RT)		
Model	GS	GE	GM	SS	GS	GE	SE	GS	SE	Total Dist.
M10.1	0.02235	0.0096	0.01598	0.01478	0.02312	0.17258	0.07416	0.09356	0.2337	2.26267
M8.1	0.019	0.00756	0.0153	0.01493	0.02435	0.14736	0.05723	0.10057	0.18695	2.28152
M8.2	0.0214	0.00885	0.01558	0.01413	0.0191	0.15994	0.04766	0.0786	0.16869	1.97597
M8.3	0.01516	0.0069	0.01387	0.02005	0.02181	0.15718	0.09139	0.09118	0.24394	2.48218
M8.4	0.01738	0.00567	0.01585	0.01493	0.02061	0.15326	0.05907	0.08493	0.18203	2.17317
M8.5	0.01908	0.00778	0.01507	0.01431	0.01819	0.16187	0.06066	0.07338	0.18544	2.0013
M7.1	0.02158	0.00928	0.01622	0.01534	0.0264	0.14367	0.05022	0.10922	0.18653	2.26475
M7.2	0.01928	0.00829	0.01518	0.01438	0.02214	0.1519	0.04803	0.08974	0.17741	1.87367
M7.3	0.01744	0.00767	0.01413	0.01392	0.0219	0.15599	0.05317	0.08815	0.17837	1.86933
M7.4	0.01857	0.00806	0.01624	0.01407	0.02187	0.14429	0.04924	0.08998	0.17694	1.92229
M7.5	0.01791	0.00987	0.01524	0.01486	0.01962	0.15251	0.05456	0.07839	0.1817	2.15058
M7.6	0.01528	0.00675	0.01382	0.01672	0.02052	0.17389	0.05394	0.08044	0.18376	2.90014
**M6.1**	**0.02312**	**0.00963**	**0.01679**	**0.01407**	**0.02051**	**0.13895**	**0.04819**	**0.08458**	**0.17723**	**1.88611**
M6.2	0.01561	0.008	0.01432	0.01518	0.01854	0.1525	0.04877	0.07507	0.1707	2.09818
M6.3	0.01323	0.00667	0.01356	0.01617	0.01895	0.16565	0.05624	0.07671	0.18724	2.6225

Table E. Out-of-sample empirical distances from posterior predictive checks

	Choice Proportions				WS Dist. (RT)			KS Dist. (RT)		
Model	GS	GE	GM	SS	GS	GE	SE	GS	SE	Total Dist.
M10.1	0.0534	0.02668	0.03917	0.07457	0.04385	0.30011	0.08903	0.16962	0.35948	4.13818
M8.1	0.05204	0.02643	0.03877	0.07455	0.04401	0.28826	0.08392	0.168	0.35133	4.14454
M8.2	0.0532	0.02668	0.03906	0.07449	0.04225	0.29657	0.0777	0.16371	0.34683	3.94099
M8.3	0.04942	0.02606	0.03809	0.0768	0.04415	0.29137	0.10524	0.17038	0.3599	4.15066
M8.4	0.05164	0.02636	0.03961	0.07468	0.04289	0.29216	0.08498	0.16388	0.34708	4.10278
M8.5	0.05151	0.02621	0.03846	0.07447	0.04297	0.29585	0.08613	0.16441	0.3492	3.99578
M7.1	0.05424	0.02745	0.04	0.07486	0.04499	0.28682	0.08011	0.17189	0.35604	4.15498
M7.2	0.05191	0.02649	0.0383	0.07517	0.0422	0.29192	0.07768	0.16093	0.34967	3.93033
M7.3	0.05207	0.02603	0.03922	0.07472	0.04323	0.29094	0.08061	0.16571	0.35008	3.94844
M7.4	0.05151	0.02725	0.03885	0.0754	0.04388	0.29	0.07913	0.16718	0.35132	3.81917
M7.5	0.04963	0.02709	0.03817	0.07459	0.04262	0.29247	0.08094	0.16392	0.34849	3.95992
M7.6	0.05061	0.02589	0.03811	0.07401	0.04299	0.30578	0.08067	0.16337	0.34761	4.8208
**M6.1**	**0.05542**	**0.02753**	**0.04007**	**0.07639**	**0.04311**	**0.28142**	**0.08023**	**0.16364**	**0.34904**	**3.77383**
M6.2	0.05014	0.02768	0.03854	0.07682	0.0432	0.29928	0.08049	0.16249	0.3459	3.88856
M6.3	0.04648	0.02659	0.03734	0.0746	0.04311	0.30706	0.08406	0.16387	0.34985	4.57465

D Comparison with alternative models

While the POMDP and RDEX models (Weigard et al., 2023) offer distinct mechanistic explanations for response inhibition, dynamic belief updating and subjective cost evaluation (POMDP) versus an accumulate-to-threshold process (RDEX), both generate comparable simulated behavior. To evaluate this, we performed a posterior predictive check comparing our POMDP framework against the best-performing RDEX variant.

To generate the simulated data, we selected three representative participants corresponding to the 95th, 50th, and 5th percentiles of the POMDP model goodness-of-fit distribution, simulating 5 task sessions per participant. For the RDEX model, we utilized the parameters for these same subjects estimated via DE-MCMC, as described in Weigard et al. (2023).

As shown in Fig J, both models successfully capture the core behavioral signatures of the task, providing adequate and comparable fits to the empirical observations. Specifically, both simulations reproduce the global outcome proportions, the right-skewed reaction time distributions for Go Success and Stop Error trials, the trial-by-trial tracking of the SSD staircases, and the inhibition performance across SSDs.

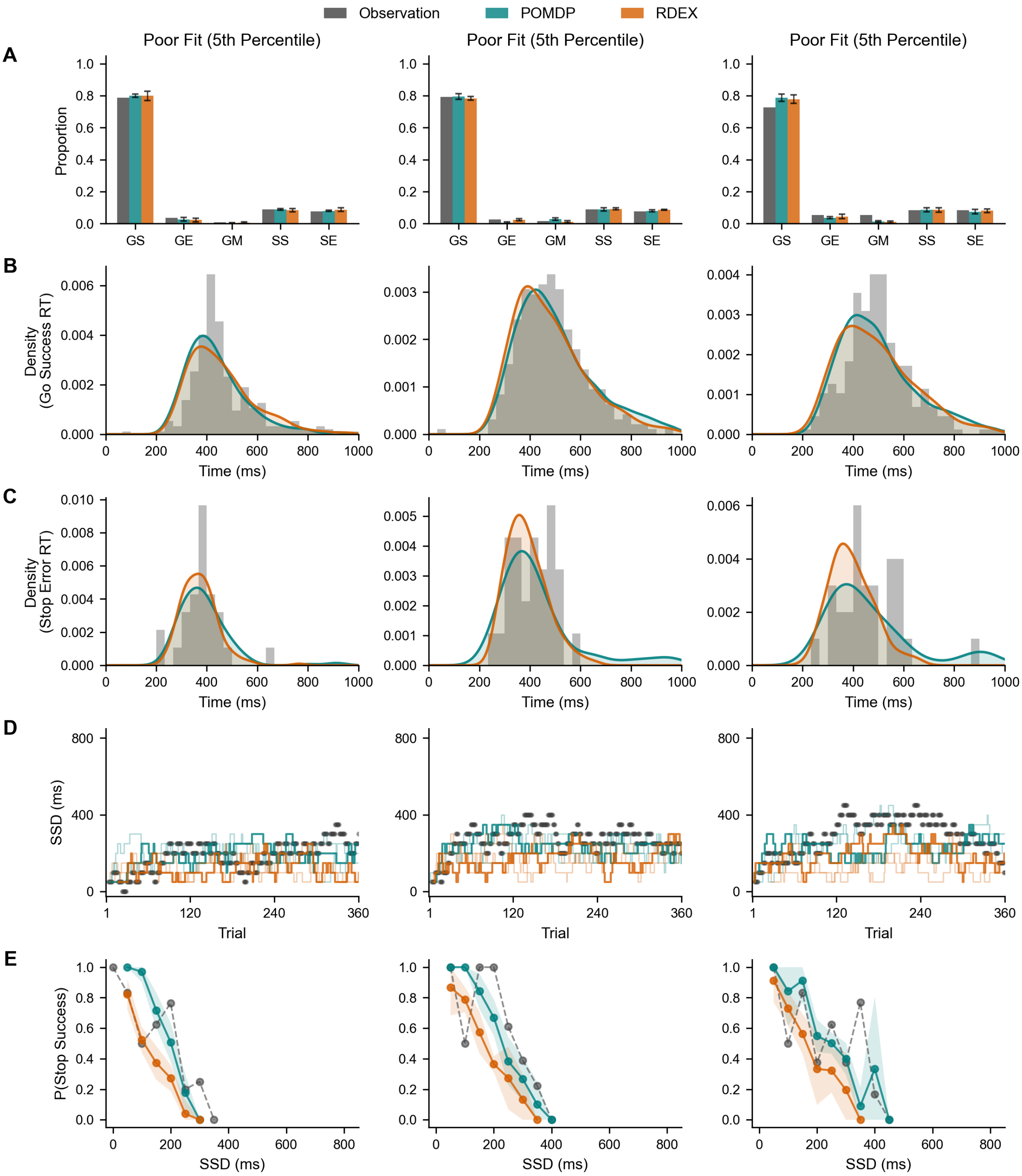

Fig J. Qualitative comparison of the POMDP and RDEX models. The columns represent three representative participants corresponding to the 95th, 50th, and 5th percentiles of the POMDP model fit distribution. The panels display simulated versus observed data across five key behavioral metrics: (A) outcome proportions, reaction time distributions for (B) Go Success and (C) Stop Error trials, (D) SSD staircase trajectories, and (E) stop accuracy across SSD. Error bars and shaded regions represent the s.e.m. Overall, although mechanistically different, both the POMDP and RDEX models yield comparable and adequate fits to the empirical data.

E Computational attributes

E.1 Aggregate distributions of computational attributes

Across the entire baseline cohort, the estimated parameters exhibit distinct aggregate profiles. The sensory parameters, go precision (χ) and stop precision (δ), are both tightly clustered. This indicates high perceptual consistency across the population, although δ features a slightly longer left tail, reflecting greater variability in stop signal processing.

In contrast, all three intrinsic cost parameters (c_{se}, c_{ge}, and c_t) exhibit distinct positive skew. This indicates that while most participants operate with relatively low subjective costs for errors and time delays, a long tail of individuals heavily penalizes these suboptimal outcomes. Finally, the non-decision time (τ) also exhibits a right-skewed distribution, averaging 5.7 steps (approximately $142\,\text{ms}$), which aligns well with known physiological bounds for visuo-motor delays.

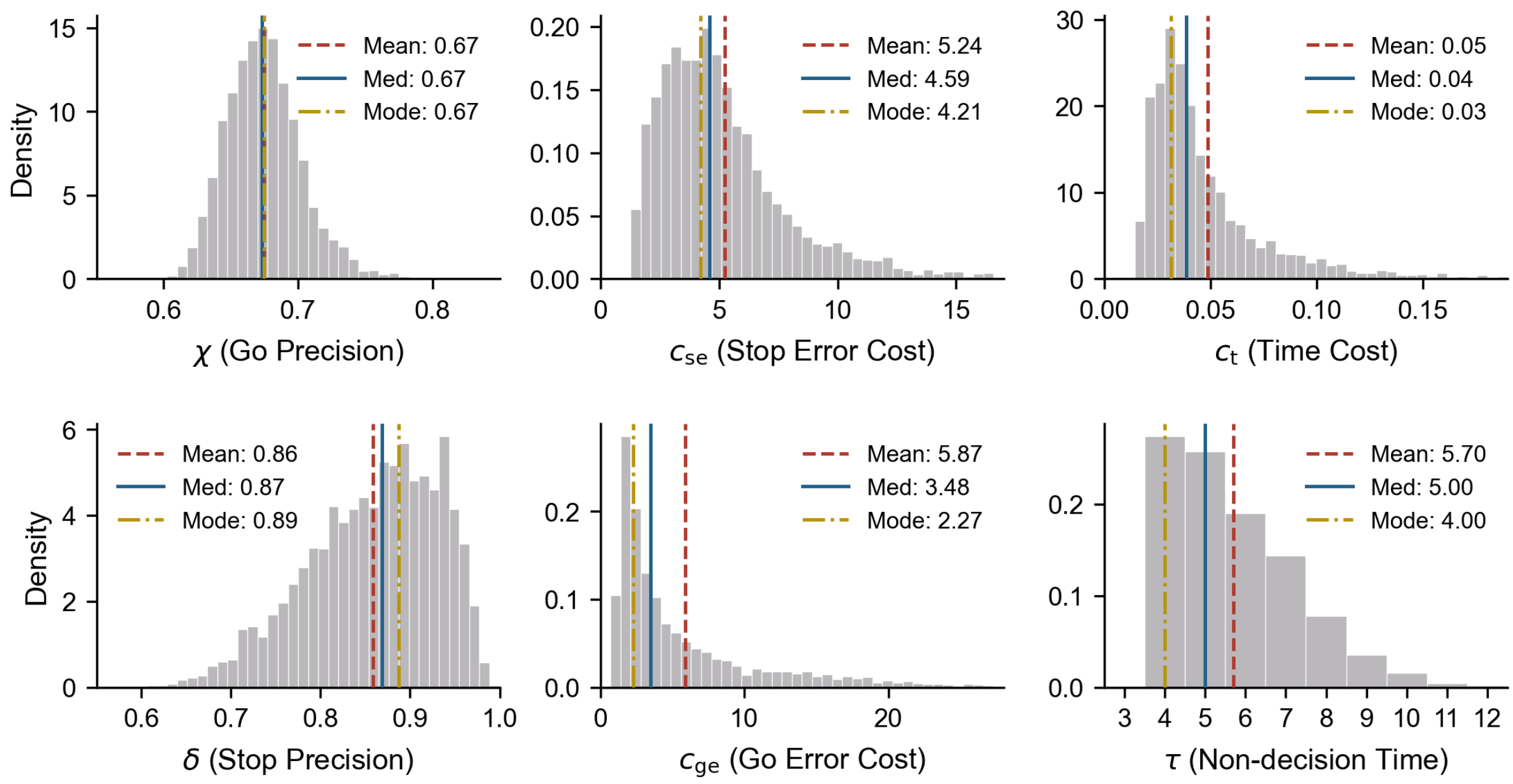

Fig K. Aggregate distributions of participant-level computational attributes. Distributions of individual point estimates across all participants ($N = 3,567$). For each participant, the point estimate is defined as the mean of 1,000 samples drawn from their respective posterior distribution. The parameters exhibit distinct distributional shapes: the sensory parameters (χ, δ) are tightly clustered, while the non-decision time (τ) and all three cost parameters ($c_{\text{se}}, c_{\text{ge}}, c_t$) display distinct positive skew. Summary statistics are provided as Mean / Median / Mode: $\chi = 0.67/0.67/0.67$; $\delta = 0.86/0.87/0.89$; $c_{\text{se}} = 5.24/4.59/4.21$; $c_{\text{ge}} = 5.87/3.48/2.27$; $c_t = 0.05/0.04/0.03$; $\tau = 5.70/5.00/4.00$.

E.2 Parameter regression and interaction analysis

To evaluate the association between ADHD scores and the estimated POMDP parameters systematically while accounting for potential confounding factors, we perform multiple linear regression analyses. For each computational parameter $\Omega \in \{\chi, \delta, c_{\text{se}}, c_{\text{ge}}, c_{\text{t}}, \tau\}$, the baseline main-effects model is formulated as follows:

$$\Omega = \iota_0 + \iota_1 \text{ADHD} + \iota_2 \text{Sex} + \iota_3 \text{IQ} + \iota_4 \text{Med} + \epsilon \tag{S16}$$

To ensure that the associations described in the main text are robust and consistent across demographic or clinical profiles, we extend this baseline model to include interaction effects. Specifically, for each

parameter, we independently add two-way interaction terms (ADHD × Sex, ADHD × IQ, and ADHD × Medication) to the main model. We also evaluate a full model that simultaneously incorporates all three interaction terms.

Statistical model parsimony and goodness-of-fit are assessed using the Bayesian Information Criterion (BIC). As detailed in Table F, the main-effects baseline model consistently generates the lowest BIC (ΔBIC $= 0$) across all six parameters. The inclusion of most individual interaction terms increases the BIC substantially (ΔBIC > 4), with the minor exception of the ADHD × Medication interaction on stop precision (δ; ΔBIC $= 1.7$). Furthermore, the full interaction models are heavily penalized (ΔBIC $\approx$ 16–24), indicating strong evidence against the added complexity.

The inclusion of interaction terms for demographic and clinical variables does not produce a significant improvement in model fit. Consequently, these interaction terms are excluded, and the main-effects model is retained as the most parsimonious and optimal. Detailed results elucidating the specific relationships between these computational attributes and clinical scores under the selected main-effects model are presented in the main text.

Table F. Supplementary: BIC Model Comparisons

Parameter	Model	*df*	BIC	ΔBIC
χ	Main	4	10058.6	0.0
	+ ADHD × Sex	5	10065.0	6.4
	+ ADHD × IQ	5	10066.0	7.4
	+ ADHD × Med	5	10063.0	4.4
	Full	7	10075.2	16.6
δ	Main	4	10151.7	0.0
	+ ADHD × Sex	5	10159.9	8.1
	+ ADHD × IQ	5	10159.8	8.0
	+ ADHD × Med	5	10153.4	1.7
	Full	7	10168.9	17.2
c_{se}	Main	4	10109.2	0.0
	+ ADHD × Sex	5	10117.4	8.2
	+ ADHD × IQ	5	10117.2	8.0
	+ ADHD × Med	5	10117.4	8.2
	Full	7	10133.5	24.3
c_{ge}	Main	4	10138.4	0.0
	+ ADHD × Sex	5	10146.0	7.7
	+ ADHD × IQ	5	10146.2	7.8
	+ ADHD × Med	5	10146.4	8.0
	Full	7	10162.0	23.6
c_t	Main	4	10124.0	0.0
	+ ADHD × Sex	5	10132.2	8.1
	+ ADHD × IQ	5	10130.6	6.5
	+ ADHD × Med	5	10131.8	7.8
	Full	7	10146.5	22.5
τ	Main	4	10144.1	0.0
	+ ADHD × Sex	5	10152.3	8.1
	+ ADHD × IQ	5	10151.1	7.0
	+ ADHD × Med	5	10152.0	7.8
	Full	7	10167.2	23.0

Note: ΔBIC = 0 indicates the preferred model.

E.3 Predictor sets comparison

To evaluate the predictive performance of the computational attributes relative to classic behavioral metrics in predicting ADHD scores, we compare three distinct predictor sets: (1) Classic metrics: SSRT, mean Go reaction time (Mean Go RT), and standard deviation of Go reaction time (Go RT SD). Notably, SSRT is derived by subtracting the mean SSD from the mean Go RT, which might be biased due to violations of the independence assumption; (2) POMDP Model: the six computational attributes; and (3) Combination: a combination of both sets. All regression models include sex, IQ, and medication status as baseline covariates.

As summarized in Table G, the POMDP Model explains slightly more variance (Adjusted $R^2 = 0.1768$) than the Classic metrics (Adjusted $R^2 = 0.1759$). Furthermore, a Likelihood Ratio Test (LRT) comparing the Combination model against the baseline confirms that incorporating the POMDP parameters significantly improves the overall model fit ($p = 0.026$). This confirms that, beyond within-trial cognitive dynamics, the computational attributes capture unique, clinically relevant variance. Despite the BIC penalty for added complexity (BIC: 9495.2 vs. 9477.8), the favorable AIC (AIC: 9433.4 vs. 9434.5) and significant LRT highlight the POMDP model's superior explanatory power for ADHD.

Table G. Regression predictor set comparison

**Predictor Set**	**Variables**	**Adjusted R^2**	**AIC**	**BIC**	**LRT p-value**
Classic metrics	SSRT, Go RT Mean, Go RT SD	0.1759	9434.5	9477.8	—
POMDP Model	POMDP Parameters	0.1768	9433.4	9495.2	—
Combination	Classic metrics + POMDP Parameters	0.1778	9432.1	9512.4	0.026

F Model-agnostic statistical analysis

Table H shows the number of subjects excluded by each quality control criterion. Importantly, the exclusion counts listed for each criterion are independent; a single subject may fail multiple criteria simultaneously. After applying all filters of experimental quality, behavioral performance, and demographic data completeness, the final valid dataset includes 3,567 baseline subjects (31.0% of the original sample).

Table H. Subject inclusion and exclusion criteria

Category	Count (N)	% of Total
Total Baseline Subjects	11,502	100.0%
*Reasons for Exclusion (Overlapping)**		
Fewer than four events for any trial type	1,392	12.1%
Evidence of box switching	188	1.6%
Task coding error (Glitch flag)	308	2.7%
More than three stop trials with 0 ms SSD	3,817	33.2%
Mean Stop error RT $>$ Correct Go RT (Race model violation)	854	7.4%
Incomplete session ($<$ 360 trials or $<$ 60 stop trials)	252	2.2%
Missing raw data file	1,299	11.3%
More than three super fast trials (RT $<$ 200 ms)	3,058	26.6%
Go missing rate $>$ 10%	1,443	12.5%
Go error rate $>$ 10%	307	2.7%
Incomplete or missing demographic data	136	1.2%
Final Valid Dataset	3,567	31.0%

Note: Subjects may be excluded for multiple reasons.

Table I outlines the distribution of ADHD scores (symptom severity, raw scores 0–14) across key demographic variables. Because the sample demonstrates inherent imbalances, particularly at higher severity levels, we employ multiple linear regression models controlling for Sex, IQ, and Medication status to rigorously isolate the specific behavioral contributions of ADHD symptoms.

Table I. Subgroup counts by Sex, IQ, and Medication across ADHD scores.

Group	ADHD Score	0	1	2	3	4	5	6	7	8	9	10	11	12	13	14	Total
	Count	1390	656	373	316	225	206	129	103	57	40	22	20	13	12	5	3567
Sex	Female	804	345	185	137	103	72	49	43	15	18	7	6	1	3	0	1788
	Male	586	311	188	179	122	134	80	60	42	22	15	14	12	9	5	1779
IQ	High IQ	464	208	101	85	67	56	29	29	13	12	8	5	4	3	1	1085
	Low IQ	417	226	124	118	87	74	58	42	24	20	8	11	4	7	3	1223
	Mid IQ	509	222	148	113	71	76	42	32	20	8	6	4	5	2	1	1259
Medication	Medicated	8	5	12	12	26	18	23	27	21	11	10	7	6	8	3	197
	Unmedicated	1382	651	361	304	199	188	106	76	36	29	12	13	7	4	2	3370

Note: IQ groups defined by sample tertiles: Low (< 17), Mid ($17 \leq$ IQ < 20), and High (≥ 20). Due to the discrete nature of the IQ scores, the resulting groups are not exactly equal in size. Medication indicates stimulant use at assessment.

We use linear regression to test how ADHD scores, Sex, IQ, and Medication predict four basic behavioral measures (Table J): mean Go success reaction time (GSRT), Stop signal reaction time (SSRT), Go success (GS) rate, and Stop success (SS) rate. Note that SSRT estimates derived from the standard race model may be biased because the ABCD task design can violate the model's independence assumption. The results show several statistically significant, yet very subtle associations. Higher ADHD scores significantly predict lower GS rates ($p < 0.001$). Among the covariates, Sex predicts GSRT and SSRT ($p < 0.01$); IQ predicts GSRT, SSRT, and GS rate ($p < 0.01$); and Medication predicts SSRT, GS rate ($p < 0.001$), and SS rate ($p < 0.05$). Most importantly, across all models, these effects are extremely small (all Partial $R^2 \leq 0.025$ and $f^2 \leq 0.026$).

Table J. Linear model results for model-agnostic behavioral metrics

Behavioral metrics	Coef.	SE	p-value	Partial R^2	f^2
**ADHD**					
GSRT	0.2521	0.4315	0.5591	0.0001	0.0001
SSRT	0.2133	0.3516	0.5440	0.0001	0.0001
GS rate	-0.0009	0.0003	0.0003***	0.0037	0.0037
SS rate	< 0.0001	0.0001	0.8196	< 0.0001	< 0.0001
**Sex (Male)**					
GSRT	-6.5722	2.0872	0.0017**	0.0028	0.0028
SSRT	-4.6760	1.7004	0.0060**	0.0021	0.0021
GS rate	0.0016	0.0013	0.2092	0.0004	0.0004
SS rate	-0.0002	0.0007	0.7160	< 0.0001	< 0.0001
**IQ**					
GSRT	-1.3156	0.2909	< 0.0001***	0.0057	0.0057
SSRT	-0.7162	0.2370	0.0025**	0.0026	0.0026
GS rate	0.0017	0.0002	< 0.0001***	0.0248	0.0255
SS rate	-0.0001	0.0001	0.2406	0.0004	0.0004
**Medication**					
GSRT	1.7456	4.9027	0.7218	< 0.0001	< 0.0001
SSRT	-12.4613	3.9941	0.0018**	0.0027	0.0027
GS rate	0.0117	0.0030	< 0.0001***	0.0044	0.0044
SS rate	0.0038	0.0016	0.0168*	0.0016	0.0016

Note: $N = 3,567$. *$p < .05$, **$p < .01$, ***$p < .001$.
