# Supplementary material for: Decomposing response inhibition: A POMDP model
Source: PLoS Comput Biol. 2026 Sep 25;22(9):e1014068. doi: 10.1371/journal.pcbi.1014068 (PMC13693991; doi:10.1371/journal.pcbi.1014068)
Supplement: Supplementary file 2 [file pcbi.1014068.s003.pdf]

Response to Reviewers

June 11, 2026

Reviewer #1

Reviewer Comment:

> The study proposed a computational framework for modeling human behavior on the Stop Signal Task (SST). The main components are a probabilistic generative model that connects stimuli to subject perception, a probabilistic inference model that models human belief updates, an optimal control algorithm to model subject action, and a simulation-based inference model to infer model parameters given subject trajectory. The framework gives insights on how individual differences on the SST arise from a set of interpretable parameters. The framework shows some predictive power for ADHD scores, indicating potential applications. I don't have major concerns about this study, but only several minor suggestions that could improve the readability of the manuscript.

Thank you very much for your helpful suggestions.

Reviewer Comment:

> The main text is overly long. I understand that due to the complexity of the proposed computational framework, the majority of the text is dedicated to explaining the technical details. Even so, I still believe the main text can be shortened to improve readability. For example, to explain the generative and inference models, there are four subsections explaining the go and stop processes, which is long, given that Figure 2 already gives a good preview of the models. The following sections have the same issue; technical details about the optimal control model and algorithm and the SBI model, should be moved to the appendix.

We apologise that the paper was so extended. We have streamlined the text with three main changes:

(1) compressing the four perceptual inference subsections into two concise sections ("The go process" and "The stop process", Page 6-9);

(2) moving the technical details of the optimal control model, including state transition probabilities, to Section A of the S1 Appendix; and

(3) relocating the implementation details of the TeSBI model and fitting algorithm to Section B of the S1 Appendix.

Since these changes are so extensive, we have not copied them into this response letter.

Reviewer Comment:

> Line 24: "In such contexts, race models relying on independence assumptions yield biased estimates of the Stop Signal Reaction Time (SSRT)." What is the bias?

The bias originates from the visual masking in the ABCD experimental design. The stop signal prematurely terminates the visual go stimulus. In the model, this truncation specifically slows the ongoing go process on stop trials. The standard race model for the SST relies on the independence assumption. It assumes the go process remains identical across all trials. When the actual go process on stop trials slows down, standard SSRT calculations become distorted. These calculations are based on the overall go reaction time. Applying them here yields confounded estimates. We have revised the manuscript to state this mechanism explicitly:

[Materials and methods (Page 3, Lines 54 - 63)] SST experimental designs are generally categorized as independent or dependent. In an independent design, the go cue remains visible when the stop signal appears. Classic formalizations, such as the independent race model, duly assume that go and stop signals are processed independently (although some neural evidence weighs against this assumption. In contrast, the ABCD study utilizes a dependent design (Figure 1). In this version, the stop signal visually

replaces, or masks, the preceding go cue. This replacement would disrupt and slow the processing of the go signal specifically on stop trials. Consequently, the core race model assumption that the go process remains identical across trial types is violated. This structural dependence confounds standard SSRT calculations.

Reviewer Comment:

> The "dependent" and "independent" SST should be stated with more clarity. "Dependent" or "independent" is about the design choice of an SST experiment; is my understanding correct? And the independent design is when there is no signal fusion between "go" and "stop" before the last readout stage of the model? Or is it about a specific SST experimental design? Or both?

Apologies for not being clearer. Yes, your understanding is correct. "Dependent" and "Independent" refer strictly to the experimental design. In an independent design, the go cue remains visible alongside the stop signal. In the ABCD design, as discussed in response to your previous comment, there is dependence because the stop signal visually masks the preceding go cue.

Classic models of the SST typically assume independence between go and stop processes (as in independent racers). However, neural evidence shows these processes interact even in independent designs. The visual masking in the dependent design forces a structural dependence. This physical overlap exacerbates the violation of the cognitive independence assumption. We have rewritten the relevant paragraph to separate the experimental design more clearly from the modeling assumptions (it is the same as above, repeated for completeness):

[Materials and methods (Page 3, Lines 54 - 63)] SST experimental designs are generally categorized as independent or dependent. In an independent design, the go cue remains visible when the stop signal appears. Classic formalizations, such as the independent race model, duly assume that go and stop signals are processed independently (although some neural evidence weighs against this assumption. In contrast, the ABCD study utilizes a dependent design (Figure 1). In this version, the stop signal visually replaces, or masks, the preceding go cue. This replacement would disrupt and slow the processing of the go signal specifically on stop trials. Consequently, the core race model assumption that the go process remains identical across trial types is violated. This structural dependence confounds standard SSRT calculations.

Reviewer Comment:

> Line 83: "We only included participants who met rigorous quality control criteria, including adherence to official ABCD performance standards." The sentence should be changed to better reflect that the criteria are not only about participants but also the experimental setup.

This is a good point. We modified the introductory sentence to clarify that the quality control criteria ensure experimental integrity (e.g., addressing hardware and coding errors) as well as valid participant performance:

[Materials and methods (Page 4, Lines 82 - 84)] We focused on baseline data from the ABCD study sample. We applied rigorous quality control criteria that evaluated both the technical experimental setup and participant task compliance.

Reviewer Comment:

> In the section "Computational phenotypes and ADHD score associations," a few things could be clearer. What are the computational phenotypes exactly referring to? I think the authors refer to all six inferred continuous parameters collectively as "phenotypes." Or is it only inverse temperature that defines phenotypes because of its wide spread? Table 4 shows the predictive power of the parameters, but the actual coefficient numbers are hard to interpret. This is arguably the most important result of this study, such that readers would like to know how good the computational model and inferred parameters are at predicting psychiatric measures (ADHD scores). To make the coefficient numbers in Table 4 more intuitive, the authors may 1) add a new plot or modify the

existing Figure 11 to show inferred parameters together with ADHD scores, and 2) add a comparison to classic metrics of the SST (e.g., stopping speed) as regressors to predict ADHD scores.

We did indeed refer to the six inferred continuous parameters collectively as "phenotypes." However, we agree that this might be confusing, and so now we refer to them exclusively as "computational attributes". We discuss them at the start of the "Computational attributes and ADHD score associations" section in the revised manuscript.

[Result (Page 20, Line 389 - 392)] Here, we define the six core continuous parameters derived from our POMDP model collectively as latent computational attributes. These attributes quantify distinct aspects of an individual's cognitive architecture, specifically capturing the characteristics of their perceptual processing and subjective valuation.

(1) We completely agree that raw regression coefficients can be unintuitive. Following your suggestion, we have updated Figure 9 to visualize the inferred parameters stratified by ADHD severity bins. This provides a much more intuitive understanding of how the computational attributes associate with clinical measures.

[Result (Page 20, Line 429 - 445)] We can illustrate these relationships by visualizing the distributions of the computational attributes stratified by ADHD scores and sex (Figure 9). As expected from the regressions, we observe population-level shifts across the clinical scores. For instance, the parameters significantly associated with ADHD, go precision (χ), stop error cost (c_{se}), go error cost (c_{ge}), and time cost (c_t), highlighted with dashed mean lines, exhibit leftward shifts as ADHD scores increase. The distributions also highlight robust sex differences in specific cost valuations. Across all ADHD severity bins, males consistently show higher c_{se} and c_t, and lower c_{ge} compared to females, maintaining a visible gap between their respective group means. However, although these shifts are highly statistically significant given the large sample size, they appear visually subtle due to the small absolute effect sizes.

Crucially, the plots reveal substantial within-group heterogeneity. The wide probability densities and the extensive overlap between distinct clinical and demographic subgroups emphasize that ADHD is computationally heterogeneous. Rather than forming a homogeneous cluster, individuals with identical clinical scores can possess vastly different underlying cognitive architectures.

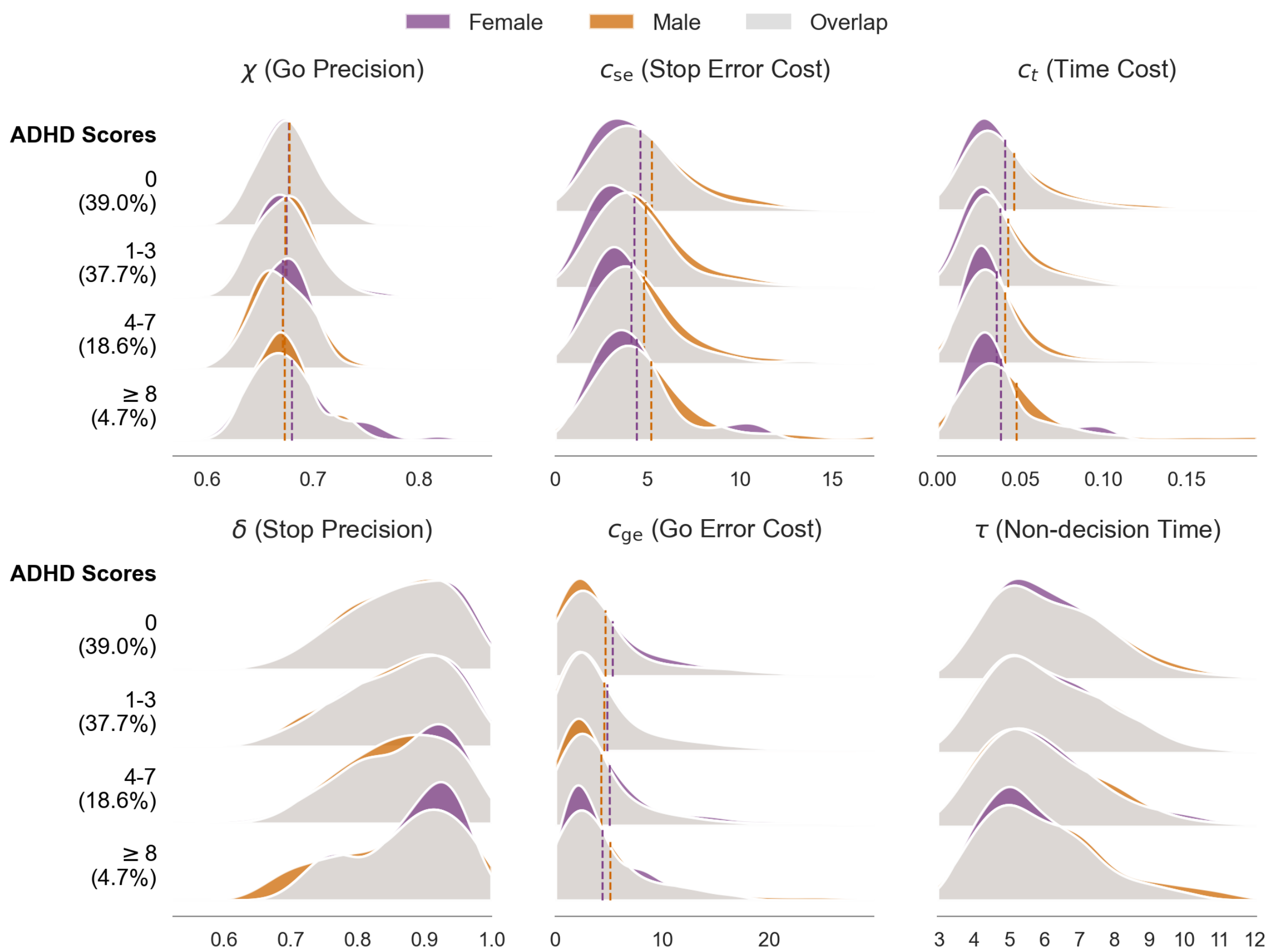

Fig 9. Distributions of participant-level computational attributes by ADHD scores and sex. Posterior distributions of the six POMDP parameters across the valid baseline sample ($N = 3,567$). Participants are categorized into four clinical bins based on their ADHD scores (0, 1-3, 4-7, ≥8). Density plots are colored by sex, with the overlapping intersection shaded in gray. Vertical dashed lines denote the group-specific means for the four parameters exhibiting significant main effects of ADHD. The visualization highlights three key features of the computational attributes: progressive mean shifts across the clinical gradient, with four parameters showing significant decreases as ADHD scores increase (go precision χ, $p < .001$; c_{se} and c_t, $p < .01$; c_{ge}, $p < .05$); robust sex differences confined to the three cost valuations (c_{se}, c_{ge}, c_t, all $p < .01$); and general individual heterogeneity, evidenced by wide probability densities and extensive overlap even between extreme clinical subgroups.

(2) This is an excellent suggestion for establishing the incremental validity of our approach. We have performed the requested comparative regression analysis. The results confirm that our computational attributes capture unique, clinically relevant information that traditional SST metrics miss. We have summarized this in the main text and detailed the full comparison in Section E.3 of S1 Appendix.

[Result (Page 20, Line 424 - 428)] Beyond mechanistic insights, POMDP parameters explain slightly more variance in ADHD scores than classic behavioral metrics after controlling for covariates. Adding these parameters to classic metrics significantly improves overall model fit, demonstrating they capture unique clinical information missed by conventional summaries (see Section E.3 of S1 Appendix).

[Section E.3 of S1 Appendix; Predictor sets comparison] To evaluate the predictive performance of the computational attributes relative to classic behavioral metrics in predicting ADHD scores, we compare three distinct predictor sets: (1) Classic metrics: SSRT, mean Go reaction time (Mean Go RT), and standard deviation of Go reaction time (Go RT SD). Notably, SSRT is derived by subtracting the mean SSD from the mean Go RT, which might be biased due to violations of the independence assumption; (2) POMDP Model: the six computational attributes; and (3) Combination: a combination of both sets. All regression models include sex, IQ, and medication status as baseline covariates.

As summarized in Table G, the POMDP Model explains slightly more variance (Adjusted $R^2 = 0.1768$)

than the Classic metrics (Adjusted $R^2 = 0.1759$). Furthermore, a Likelihood Ratio Test (LRT) comparing the Combination model against the baseline confirms that incorporating the POMDP parameters significantly improves the overall model fit ($p = 0.026$). This confirms that, beyond within-trial cognitive dynamics, the computational attributes capture unique, clinically relevant variance. Despite the BIC penalty for added complexity (BIC: 9495.2 vs. 9477.8), the favorable AIC (AIC: 9433.4 vs. 9434.5) and significant LRT highlight the POMDP model's superior explanatory power for ADHD.

Table G. Regression predictor set comparison

Predictor Set	Variables	Adjusted R^2	AIC	BIC	LRT p-value
Classic metrics	SSRT, Go RT Mean, Go RT SD	0.1759	9434.5	9477.8	—
POMDP Model	POMDP Parameters	0.1768	9433.4	9495.2	—
Combination	Classic metrics + POMDP Parameters	0.1778	9432.1	9512.4	0.026

Reviewer #2

Reviewer Comment:

> This is a technically rigorous manuscript that makes a genuine methodological contribution by combining POMDP modeling with transformer-based simulation inference for large-scale behavioral data. The model is carefully formulated, the inference pipeline is well-validated, and the theoretical properties are comprehensively illustrated. However, the clinical findings are weak and risk being overclaimed, several modeling choices need better justification, and a number of errors and inconsistencies need correction. I recommend minor revision, with particular attention to: (1) tempering the clinical narrative and reporting effect sizes, (2) justifying the fixed/free parameter structure, (3) addressing the stop-parameter identifiability concern, (4) fixing the sample-size discrepancy and broken reference, and (5) providing code for review.

Thank you for this assessment and your very helpful comments. We have followed all your suggestions.

Reviewer Comment:

> Major Points
>
> 1. ADHD effect sizes are missing and likely trivial. Table 4 reports only unstandardized coefficients with Wald SEs. With N = 5,114, statistical significance is nearly guaranteed for any nonzero effect. The authors should report standardized effect sizes (partial R^2, Cohen's f^2) for all ADHD associations. Without these, the reader cannot evaluate the clinical relevance of the findings. The abstract and framing should be adjusted to match the "rather weak" associations (line 570).

It is a very good point that reporting standardized effect sizes is crucial for evaluating clinical relevance in large-scale datasets. Thus, we have now implemented the following changes:

(1) We standardized all continuous variables prior to regression. The updated Table 4 now reports standardized coefficients, partial R^2, and Cohen's f^2 for all predictors. (Note: The sample size is now N=3,567 following stricter quality control, as detailed in our other responses).

(2) As the reviewer anticipated, the effect sizes for ADHD associations are small. Importantly, our updated analysis reveals two key patterns:

   (a) The effect sizes for ADHD traits are in the same range as established covariates Sex and IQ (all partial $R^2 < 0.015$, Cohen's $f^2 < 0.015$).

   (b) All predictors (ADHD traits, Sex and IQ) show a comparable effect size profile across the parameters, with their strongest relative influence on the stop error cost.

(3) We have revised both the Abstract and the Results sections to acknowledge these "uniformly small" effect sizes explicitly, ensuring we do not overstate the strength of the clinical associations:

[Abstract] We identified distinct latent computational attributes associated with scores on ADHD questionnaires. Controlling for sex, IQ, and medication status, children with higher ADHD scores exhibited subtle but robust shifts in computational attributes. They showed a blunted sensitivity to the costs of both errors and time, alongside slightly diminished directional precision.

[Result (Page 21, Line 420 - 422)] Notably, while statistically robust, absolute effect sizes remain generally small (partial $R^2 < 0.015$, Cohen's $f^2 < 0.015$). However, ADHD characteristics and established covariates (Sex and IQ) exhibit comparable, parameter-specific effect size profiles.

Table 4. Parameter Regression Coefficients and Effect Sizes

Parameters	β	SE	p-value	Partial R^2	f^2
**ADHD**					
χ	-0.0681	0.0182	0.0002***	0.0039	0.0039
δ	-0.0038	0.0184	0.8351	< 0.0001	< 0.0001
c_{se}	-0.0533	0.0183	0.0036**	0.0024	0.0024
c_{ge}	-0.0427	0.0184	0.0201*	0.0015	0.0015
c_t	-0.0498	0.0183	0.0067**	0.0021	0.0021
τ	-0.0256	0.0184	0.1635	0.0005	0.0005
**Sex (Male)**					
χ	-0.0244	0.0336	0.4684	0.0001	0.0001
δ	-0.0435	0.0340	0.2017	0.0005	0.0005
c_{se}	0.2143	0.0338	< 0.0001***	0.0111	0.0113
c_{ge}	-0.1094	0.0340	0.0013**	0.0029	0.0029
c_t	0.1584	0.0339	< 0.0001***	0.0061	0.0061
τ	0.0006	0.0340	0.9866	< 0.0001	< 0.0001
**IQ**					
χ	0.1455	0.0166	< 0.0001***	0.0211	0.0216
δ	-0.0374	0.0168	0.0264*	0.0014	0.0014
c_{se}	0.0362	0.0167	0.0306*	0.0013	0.0013
c_{ge}	0.0242	0.0168	0.1496	0.0006	0.0006
c_t	0.0492	0.0168	0.0033**	0.0024	0.0024
τ	0.0534	0.0168	0.0015**	0.0028	0.0028
**Medication**					
χ	0.2782	0.0789	0.0004***	0.0035	0.0035
δ	-0.0793	0.0799	0.3210	0.0003	0.0003
c_{se}	0.1109	0.0794	0.1628	0.0005	0.0005
c_{ge}	0.1618	0.0798	0.0426*	0.0012	0.0012
c_t	0.0706	0.0796	0.3752	0.0002	0.0002
τ	-0.0655	0.0798	0.4122	0.0002	0.0002

Note: $N = 3,567$. $^{*}p < .05$, $^{**}p < .01$, $^{***}p < .001$.

Reviewer Comment:

> 2. Fixed vs. free parameter choices need justification. Fixing $\lambda = 0.2$ (hazard rate) and $\tilde{r}^{\nu} = 1/6$ (stop prior) while freeing six other parameters is a consequential decision that is inadequately motivated ("based on previous work [21] and pilot testing," line 362). Fig 9 demonstrates that $\tilde{r}^{\nu}$ profoundly affects behavior. Meanwhile, Yu & Dayan (2005; Ref [20]) showed participants adaptively track stop probability, suggesting λ and $\tilde{r}^{\nu}$ could absorb variance currently attributed to other parameters. A sensitivity analysis varying the fixed values, or a formal model comparison, would strengthen the paper.

We appreciate that fixing these parameters requires more rigorous justification than we previously provided. We therefore completely overhauled our parameter selection process and added a sensitivity analysis.

(1) Instead of relying on pilot testing, we updated the fixed parameters to align with the true generative statistics of the ABCD version of the task:

   (1) Hazard Rate (λ): We adjusted lambda from 0.2 to 0.1 to reflect the task's temporal structure (the mean SSD is approximately 250 ms, equivalent to 10 time steps; therefore, we set lambda = 1/10).

   (2) Stop Prior ($\tilde{r}^{\nu}$): We fixed the stop prior to 1/6 to reflect the exact global task design (60 stop trials out of 360 total).

(2) To test whether these parameters could be freed without absorbing variance from one another, we conducted a rigorous, iterative step-down model comparison evaluating 15 variants (detailed in the Model selection and validation of the main text; and Section C of S1 Appendix). Starting from an unconstrained full model, we systematically fixed parameters with poor recovery ($r < 0.65$) to specific posterior quantiles, repeating this reduction until all remaining free parameters achieved robust identifiability. Based on posterior predictive checks, we selected Model M6.3 as our primary model because it generates the lowest aggregate behavioral distance among the fully identifiable variants. In M6.3, four parameters are fixed (go/stop null, go missing cost, and inverse temperature), leaving six highly informative parameters free (go/stop precision, non-decision time, and stop error, go error, and time costs). Crucially, unlike the model in our original manuscript, this systematically reduced model guarantees that all free parameters are robustly identifiable (all recovery coefficients $r > 0.65$).

(3) While we agree participants track across-trial stop probabilities (Shenoy and Yu, 2011), the ABCD task's fixed 1/6 stop ratio rather restricts the fluctuations in this prior. As our new sensitivity analysis (Section A.6 of S1 Appendix) confirms, variations within this relatively narrow range minimally impact our estimates. Fixing $\tilde{r}^{\nu}$ therefore allows us to isolate within-trial dynamics from across-trial noise. We have revised the manuscript as:

Discussion (Page 24, Line 553 - 559) However, because the 1/6 stop ratio in the ABCD task restricts the fluctuation of this prior, fixing it to 1/6 allows us to focus on within-trial dynamics. As shown in our sensitivity analysis (Section A.6 of S1 Appendix), variations within this restricted range generate small but observable shifts in performance; thus, fixing the prior simplifies the current model. This approach establishes stable within-trial parameters first, allowing future extensions to free the stop prior and investigate across-trial effects.

[Section A.6 of S1 Appendix; Stop prior sensitivity analysis] In the ABCD task, stop trials account for only 1/6 of the total trials (60/360), which limits the natural fluctuation of participants' across-trial stop expectation ($\tilde{r}^{\nu}$). As shown in Figure F, simulating the POMDP model across a realistic range of prior probabilities (0.05 to 0.30) generates small but observable shifts in performance, such as slight increases in Go reaction times, as well as in Stop trial accuracy and error reaction times. Given this restricted range, we fixed $\tilde{r}^{\nu}$ to 1/6 for the current study to simplify the model and focus on estimating within-trial dynamics. This step allows us to establish stable within-trial parameters first, which can then serve as a baseline for future work to free the stop prior and investigate across-trial learning effects.

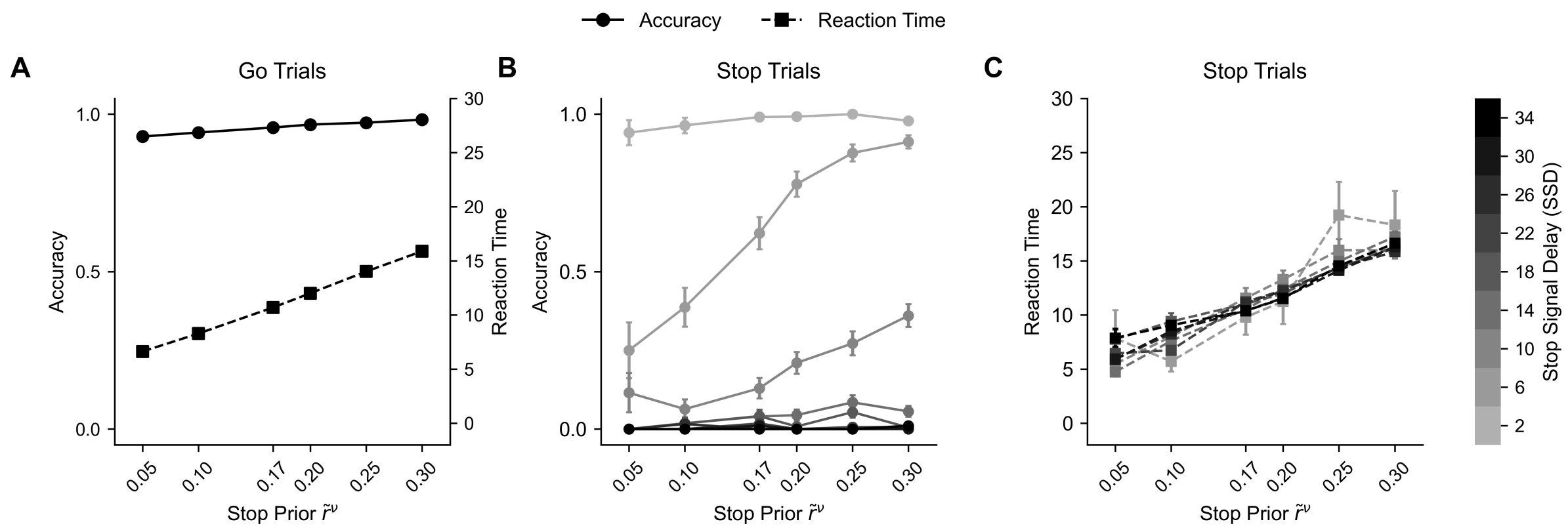

Fig F. Sensitivity of POMDP behavioral metrics to stop trial priors. Simulated outputs across varying priors ($\tilde{r}^{\nu}$), centered around the ABCD task baseline (1/6). (A) Go accuracy (solid/circles; left axis) and reaction times (dashed/squares; right axis). (B-C) Stop accuracy and reaction times across Stop Signal Delays (SSD: 2–34, grayscale). Error bars denote standard error of the mean (s.e.m.).

Reviewer Comment:

> 3. Stop-parameter recovery is moderate — implications for null ADHD findings. Recovery of δ (r = 0.67) and δ' (r = 0.60) is substantially lower than for go-related parameters. The null ADHD association with stop-signal perceptual parameters could therefore reflect estimation noise rather than a true absence of effect. This should be explicitly discussed, as stop-signal sensitivity deficits are a plausible ADHD mechanism (cf. Weigard et al., 2019; Ref [9]).

We agree that the moderate parameter recovery in our previous draft could have obscured true perceptual deficits, which are a highly plausible mechanism in ADHD, as indeed demonstrated by Weigard et al. (2019). To address this, we have addressed recovery head-on, and now also explicitly discuss the implications of our updated findings:

(1) As detailed in our response to the previous comment, we implemented a rigorous 15-variant step-down model selection procedure. By systematically fixing unidentifiable parameters, our newly selected model ensures robust recovery for all remaining free parameters. Specifically, the parameter recovery for stop precision is now robust and reliable (r = 0.66). We have revised the manuscript as:

> [Section C.3 of S1 Appendix; Parameter recovery analysis] To ensure robustness, we apply a criterion requiring $r > 0.65$ for all estimated parameters (indicated by checkmarks in the Valid column). As Table C shows, while increasing the number of free parameters enhances model flexibility to fit the data, it also introduces severe parameter unidentifiability. Our step-down procedure effectively resolves this issue. In the final selected model (M6.1), all six free parameters strictly satisfy this identifiability criterion: go precision ($r = 0.89$), stop precision ($r = 0.72$), non-decision time ($r = 0.83$), stop error cost ($r = 0.81$), time cost ($r = 0.82$), and go error cost ($r = 0.72$).

Table C: Parameter recovery correlations across model variants

**Model**	χ	δ	τ	c_{se}	c_t	c_{ge}	c_{gm}	χ'	δ'	φ	**Valid**
M10.1	0.7338	0.52576	0.76639	0.71686	0.77255	0.67475	0.25241	0.68363	0.28484	0.23538	
M8.1	0.78803	0.67084	0.76253	0.72547	0.64495	0.65898	0.19468	0.56529	—	—	
M8.2	0.75792	0.63352	0.75234	0.72376	0.68564	0.6542	0.18572	0.58766	—	—	
M8.3	0.78363	0.65966	0.75261	0.74255	0.69466	0.65715	0.25048	0.5983	—	—	
M8.4	0.77669	0.67832	0.78664	0.7536	0.66689	0.65421	0.20505	0.58863	—	—	
M8.5	0.76724	0.62577	0.7137	0.73714	0.67105	0.63331	0.26696	0.58201	—	—	
M7.1	0.77864	0.73923	0.78226	0.8094	0.74747	0.56983	—	0.66981	—	—	
M7.2	0.74035	0.74885	0.80225	0.82987	0.76128	0.60295	—	0.66004	—	—	
M7.3	0.77628	0.75358	0.78879	0.82022	0.7603	0.6681	—	0.57688	—	—	
M7.4	0.78577	0.68973	0.69869	0.81331	0.73715	0.59321	0.24727	—	—	—	
M7.5	0.80723	0.70444	0.71446	0.85211	0.78155	0.61792	0.29937	—	—	—	
M7.6	0.78866	0.70419	0.70713	0.79195	0.76916	0.57304	0.19798	—	—	—	
**M6.1**	**0.89382**	**0.72778**	**0.83096**	**0.81072**	**0.82478**	**0.72995**	—	—	—	—	✓
M6.2	0.88438	0.69043	0.84585	0.80845	0.80418	0.71801	—	—	—	—	✓
M6.3	0.89813	0.70072	0.81195	0.76931	0.76785	0.70143	—	—	—	—	✓

(2) With the parameter recovery issue resolved, we re-evaluated the ADHD associations (now Table 4). While individuals with higher ADHD scores do exhibit lower stop precision (-0.0286), this association remains statistically non-significant ($p = 0.121$). Instead, the model reveals that the variance is significantly and robustly captured by altered subjective cost valuations, specifically lower stop error cost, go error cost and time cost.

(3) We completely agree that stop-signal perceptual deficits are a highly plausible mechanism, as demonstrated by Weigard et al. (2019). Following your suggestion, we now explicitly discuss this. Specifically, we tentatively hypothesize that within a unified computational framework like the POMDP, what is traditionally interpreted as a pure perceptual deficit might be partially driven by top-down alterations in subjective cost valuation (Table 4): stop error cost (-0.0711), go error cost (-0.0517) and time cost (-0.0615). We have revised the manuscript as:

> [Discussion (Page 24, Line 517 - 523)] Crucially, our computational perspective offers new clinical insights. While prior studies have highlighted stop-signal perceptual deficits as a plausible mechanism underlying ADHD (Weigard et al., 2019), our findings tentatively suggest an alternative perspective (albeit remembering the very small effect sizes). Within a unified framework like the POMDP, we hypothesize that a substantial portion of the behavioral variance traditionally attributed to pure perceptual impairments might actually stem from top-down alterations in how individuals subjectively weigh the costs of errors and delays.

Due to length constraints, we only highlight the specific results above. We kindly direct the reviewer to Section C of S1 Appendix for the full text and detailed tables.

Reviewer Comment:

> 4. Non-decision time omission confounds parameter interpretation. Without a non-decision time component, the model may attribute motor/encoding latency to Go precision (χ) or inverse temperature (φ). This is especially relevant to the ADHD findings, since ADHD has been associated with longer non-decision times in DDM-based analyses of the same ABCD task (Weigard et al., 2023; Ref [42]). This limitation needs more than a one-sentence acknowledgment.

Thank you for this suggestion. We added a non-decision time parameter (τ) to our POMDP model and re-fit the data. Our updated results (Table 4) show a positive trend between ADHD scores and longer non-decision times, which is consistent with DDM findings from Weigard et al. (2023). However, this association does not reach statistical significance. This is likely because significant variance in non-decision time is captured by the included covariates (sex, IQ, and medication), combined with the limited clinical ADHD severity in the ABCD study. Following your suggestion, we have now added this context to the Discussion section:

[Discussion (Page 24, Line 521 - 526)] Similarly, while recent DDM-based analyses of the ABCD task link ADHD to longer non-decision times (Weigard et al., 2023), we observed only a non-significant negative trend. This divergence likely arises because our included covariates (sex, IQ, and medication) captured significant variance in non-decision time, combined with the limited clinical ADHD severity in the ABCD study.

Reviewer Comment:

> 5. No model comparison is provided. The POMDP is positioned as superior to race models for the dependent ABCD design, but this is asserted rather than demonstrated. Even a qualitative PPC comparison against a fitted RDEX-ABCD or DDM model would substantiate the claim.

We did not intend to claim that the POMDP was better in general as a way of fitting the task – just that we had structurally adapted it to the exigencies of the particular version of the SST in the ABCD task. The reviewer is quite right to point to equivalent possible adjustments to the standard RACE model – notably the RDEX-ABCD model (Weigard et al., 2023). We therefore include a qualitative comparison in the appendix (with a note in the discussion). In fact, the model fits are rather comparable – which points to an interesting direction for the future in mapping the part-computational and part-algorithmic parameters of RDEX-ABCD to the explicit costs and sensitivities of the POMDP model.

[Discussion (Page 24, Line 512 - 514)] Section D of the S1 Appendix provides a qualitative comparison between our value-based mechanism and a more standard accumulate-to-bound architecture (RDEX-ABCD), a race model adapted to the peculiarities of the ABCD task (Weigard et al., 2023). The models fit comparably well, opening an important question for the future in mapping their rather different parameterizations onto each other.

[Section D of S1 Appendix; Comparison with alternative models] While the POMDP and RDEX models (Weigard et al., 2023) offer distinct mechanistic explanations for response inhibition, dynamic belief updating and subjective cost evaluation (POMDP) versus an accumulate-to-threshold process (RDEX), both generate comparable simulated behavior. To evaluate this, we performed a posterior predictive check comparing our POMDP framework against the best-performing RDEX variant.

To generate the simulated data, we selected three representative participants corresponding to the 95th, 50th, and 5th percentiles of the POMDP model goodness-of-fit distribution, simulating 5 task sessions per participant. For the RDEX model, we utilized the parameters for these same subjects estimated via DE-MCMC, as described in Weigard et al. (2023).

As shown in Figure J, both models successfully capture the core behavioral signatures of the task, providing adequate and comparable fits to the empirical observations. Specifically, both simulations reproduce the global outcome proportions, the right-skewed reaction time distributions for Go Success and Stop Error trials, the trial-by-trial tracking of the SSD staircases, and the inhibition performance across SSDs.

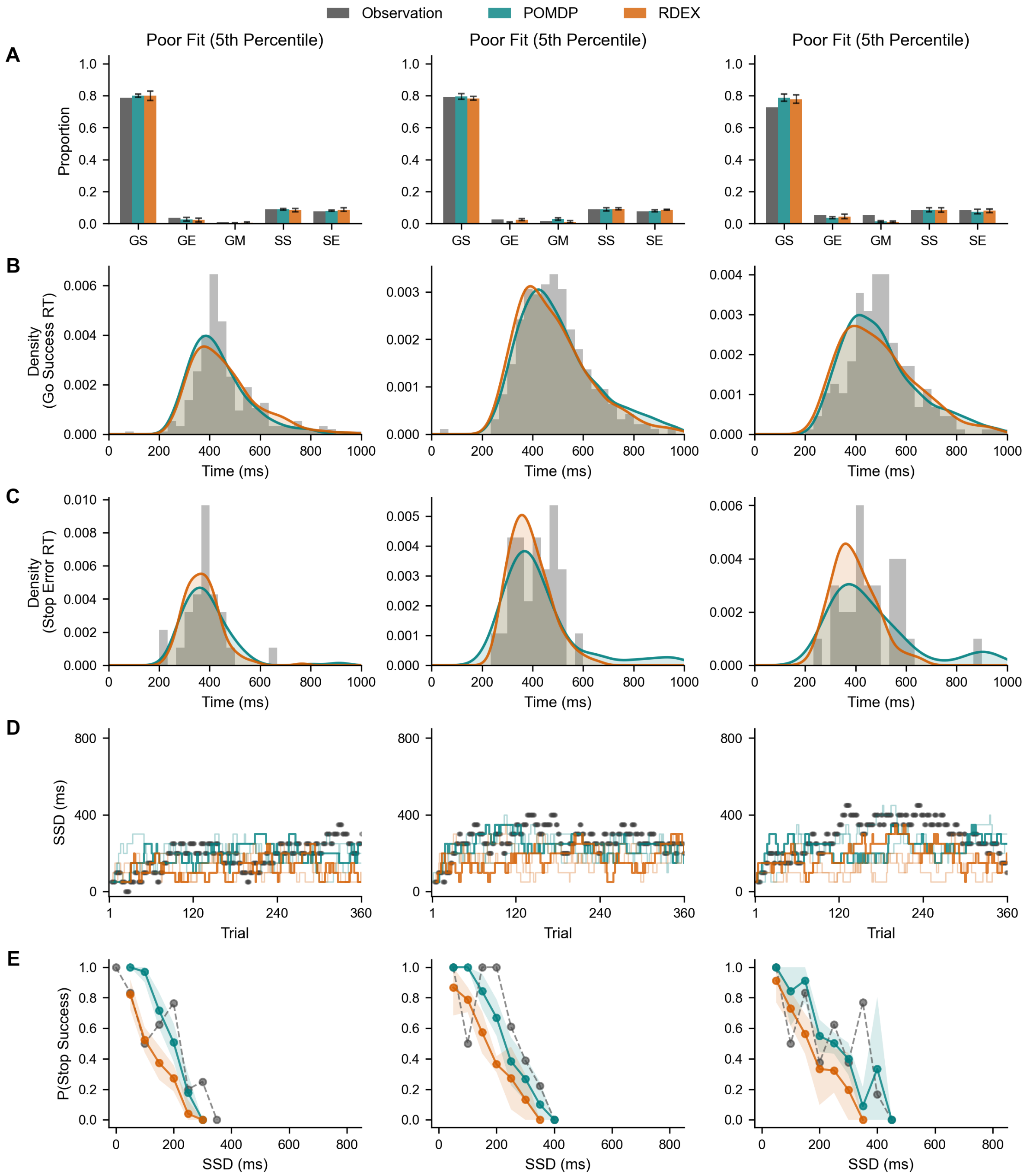

Fig J. Qualitative comparison of the POMDP and RDEX models. The columns represent three representative participants corresponding to the 95th, 50th, and 5th percentiles of the POMDP model fit distribution. The panels display simulated versus observed data across five key behavioral metrics: (A) outcome proportions, reaction time distributions for (B) Go Success and (C) Stop Error trials, (D) SSD staircase trajectories, and (E) stop accuracy across SSD. Error bars and shaded regions represent the s.e.m. Overall, although mechanistically different, both the POMDP and RDEX models yield comparable and adequate fits to the empirical data.

Reviewer Comment:

Minor Points:

(1) Sample size discrepancy: Main text says N = 5,114; Supplementary Table B says N = 5,144.

Which is correct?

(2)Broken reference: Line 633 contains "[?]" — a missing LaTeX citation for time perception noise.

(3) Wrong symbol (line 429): Text says stop belief δ_t^ν but should be σ_t^ν? δ is the stop precision parameter.

(4) Fig 10 caption: "highlighted (teal) model's adaptive behavior" is missing a word.

(5) Typo (line 310): "modeling the staircase using in ABCD" to "used in ABCD."

(6) Inconsistent naming: "Section B.2 of Appendix S1" (line 544) vs. "S1 Appendix" elsewhere.

We are very grateful for the careful reading and for catching these typographical and formatting errors. We have corrected all of them in the revised manuscript as follows:

(1) Sample size issue: Following stricter data quality criteria (detailed in our earlier responses), the validated baseline sample size is now updated to N = 3,567. This has been consistently corrected throughout the main text and appendix.

(2) Broken reference (Line 633): The LaTeX compilation artifact has been resolved, and Rakitin et al. (1998) is now correctly cited.

(3) Symbol error (Line 429): The symbol for stop belief has been corrected from δ_t^ν to σ_t^ν.

(4) Figure 10 caption: The missing word has been added, now reading: "...with three individual simulations (teal) highlighting the model's adaptive behavior."

(5) Typo (Line 310): The grammatical error has been corrected to read: "regulated by an adaptive staircase procedure used in ABCD."

(6) Inconsistent naming: We have standardized all appendix references to "S1 Appendix" throughout the manuscript.

Reviewer Comment:

The authors said that "all code used for the POMDP modeling, parameter inference, and statistical analyses will be made publicly available on the Open Science Framework" (line 693), but no repository link or deposited code is provided with the current submission.

We thank the reviewer for pointing this out. We apologize for the omission in the initial submission. We have now updated the manuscript to include the direct link to the publicly available repository on the Open Science Framework (OSF). The revised text in the Data and code availability section now reads as follows:

[Data and code availability (Page 26,Line 609 - 612)] All custom code used for the POMDP modeling, parameter inference, and statistical analyses is publicly available on the Open Science Framework (OSF) at https://doi.org/10.17605/OSF.IO/UQNCE and on GitHub at https://github.com/wenting-wang/sst-pomdp.
