# Supplementary material for: Decomposing response inhibition: A POMDP model
Source: PLoS Comput Biol. 2026 Sep 25;22(9):e1014068. doi: 10.1371/journal.pcbi.1014068 (PMC13693991; doi:10.1371/journal.pcbi.1014068)
Supplement: Peer review history

# Peer review: Decision Letter 0

**DOI:** 10.1371/journal.pcbi.1014068.r001

**Authors:** Andrea E. Martin, Jian Liu

19 Apr 2026

PCOMPBIOL-D-26-00444

Decomposing response inhibition: a POMDP model

PLOS Computational Biology

Dear Dr. Wang,

Thank you for submitting your manuscript to PLOS Computational Biology. After careful consideration, we feel that it has merit but does not fully meet PLOS Computational Biology's publication criteria as it currently stands. Therefore, we invite you to submit a revised version of the manuscript that addresses the points raised during the review process.

Please submit your revised manuscript by Jun 19 2026 11:59PM. If you will need more time than this to complete your revisions, please reply to this message or contact the journal office at ploscompbiol@plos.org. When you're ready to submit your revision, log on to https://www.editorialmanager.com/pcompbiol/ and select the 'Submissions Needing Revision' folder to locate your manuscript file.

Please include the following items when submitting your revised manuscript:

* A letter that responds to each point raised by the editor and reviewer(s). You should upload this letter as a separate file labeled 'Response to Reviewers'. This file does not need to include responses to formatting updates and technical items listed in the 'Journal Requirements' section below.

* A marked-up copy of your manuscript that highlights changes made to the original version. You should upload this as a separate file labeled 'Revised Manuscript with Track Changes'.

* An unmarked version of your revised paper without tracked changes. You should upload this as a separate file labeled 'Manuscript'.

If you would like to make changes to your financial disclosure, competing interests statement, or data availability statement, please make these updates within the submission form at the time of resubmission. Guidelines for resubmitting your figure files are available below the reviewer comments at the end of this letter.

We look forward to receiving your revised manuscript.

Kind regards,

Jian Liu

Academic Editor

PLOS Computational Biology

Andrea E. Martin

Section Editor

PLOS Computational Biology

**Additional Editor Comments:**

The manuscript has clear merit. However, some additional clarifications are needed to improve overall readability. Please revise the text accordingly.

**Journal Requirements:**

If the reviewer comments include a recommendation to cite specific previously published works, please review and evaluate these publications to determine whether they are relevant and should be cited. There is no requirement to cite these works unless the editor has indicated otherwise.

1) Please ensure that the CRediT author contributions listed for every co-author are completed accurately and in full.

At this stage, the following Authors/Authors require contributions: Wenting Wang, Peter Dayan, and Tobias Kaufmann. Please ensure that the full contributions of each author are acknowledged in the "Add/Edit/Remove Authors" section of our submission form.

The list of CRediT author contributions may be found here: https://journals.plos.org/ploscompbiol/s/authorship#loc-author-contributions

2) We ask that a manuscript source file is provided at Revision. Please upload your manuscript file as a .doc, .docx, .rtf or .tex. If you are providing a .tex file, please upload it under the item type u2018LaTeX Source Fileu2019 and leave your .pdf version as the item type u2018Manuscriptu2019.

3) Thank you for including an Ethics Statement for your study. Please include:

i) The approval number(s), or a statement that approval was granted by the named board(s)

ii) A statement that formal consent was obtained (must state whether verbal/written) OR the reason consent was not obtained (e.g. anonymity). NOTE: If child participants, the statement must declare that formal consent was obtained from the parent/guardian.].

4) Please upload all main figures as separate Figure files in .tif or .eps format. For more information about how to convert and format your figure files please see our guidelines:

https://journals.plos.org/ploscompbiol/s/figures

5) Please ensure that all Figure files have corresponding citations and legends within the manuscript. Currently, Figure 1 in your submission file inventory does not have an in-text citation. If the figure is no longer to be included as part of the submission, please remove it from the file inventory.

6) In the online submission form, you indicated that your data will be submitted to a repository upon acceptance. We strongly recommend all authors deposit their data before acceptance, as the process can be lengthy and hold up publication timelines. Please note that, though access restrictions are acceptable now, your entire minimal dataset will need to be made freely accessible if your manuscript is accepted for publication. This policy applies to all data except where public deposition would breach compliance with the protocol approved by your research ethics board. If you are unable to adhere to our open data policy, please kindly revise your statement to explain your reasoning and we will seek the editor's input on an exemption.

7)  Please ensure that the funders and grant numbers match between the Financial Disclosure field and the Funding Information tab in your submission form. Note that the funders must be provided in the same order in both places as well.

**Reviewers' comments:**

Reviewer's Responses to Questions

**Comments to the Authors:**

**Please note here if the review is uploaded as an attachment.**

Reviewer #1: The study proposed a computational framework for modeling human behavior on the Stop Signal Task (SST). The main components are a probabilistic generative model that connects stimuli to subject perception, a probabilistic inference model that models human belief updates, an optimal control algorithm to model subject action, and a simulation-based inference model to infer model parameters given subject trajectory. The framework gives insights on how individual differences on the SST arise from a set of interpretable parameters. The framework shows some predictive power for ADHD scores, indicating potential applications.

I don't have major concerns about this study, but only several minor suggestions that could improve the readability of the manuscript.

The main text is overly long. I understand that due to the complexity of the proposed computational framework, the majority of the text is dedicated to explaining the technical details. Even so, I still believe the main text can be shortened to improve readability. For example, to explain the generative and inference models, there are four subsections explaining the go and stop processes, which is long, given that Figure 2 already gives a good preview of the models. The following sections have the same issue; technical details about the optimal control model and algorithm and the SBI model, should be moved to the appendix.

Line 24: "In such contexts, race models relying on independence assumptions yield biased estimates of the Stop Signal Reaction Time (SSRT)." What is the bias?

The "dependent" and "independent" SST should be stated with more clarity. "Dependent" or "independent" is about the design choice of an SST experiment; is my understanding correct? And the independent design is when there is no signal fusion between "go" and "stop" before the last readout stage of the model? Or is it about a specific SST experimental design? Or both?

Line 83: "We only included participants who met rigorous quality control criteria, including adherence to official ABCD performance standards." The sentence should be changed to better reflect that the criteria are not only about participants but also the experimental setup.

In the section "Computational phenotypes and ADHD score associations," a few things could be clearer. What are the computational phenotypes exactly referring to? I think the authors refer to all six inferred continuous parameters collectively as "phenotypes." Or is it only inverse temperature that defines phenotypes because of its wide spread? Table 4 shows the predictive power of the parameters, but the actual coefficient numbers are hard to interpret. This is arguably the most important result of this study, such that readers would like to know how good the computational model and inferred parameters are at predicting psychiatric measures (ADHD scores). To make the coefficient numbers in Table 4 more intuitive, the authors may 1) add a new plot or modify the existing Figure 11 to show inferred parameters together with ADHD scores, and 2) add a comparison to classic metrics of the SST (e.g., stopping speed) as regressors to predict ADHD scores.

Reviewer #2: This is a technically rigorous manuscript that makes a genuine methodological contribution by combining POMDP modeling with transformer-based simulation inference for large-scale behavioral data. The model is carefully formulated, the inference pipeline is well-validated, and the theoretical properties are comprehensively illustrated. However, the clinical findings are weak and risk being overclaimed, several modeling choices need better justification, and a number of errors and inconsistencies need correction. I recommend minor revision, with particular attention to: (1) tempering the clinical narrative and reporting effect sizes, (2) justifying the fixed/free parameter structure, (3) addressing the stop-parameter identifiability concern, (4) fixing the sample-size discrepancy and broken reference, and (5) providing code for review.

Major Points

1. ADHD effect sizes are missing and likely trivial. Table 4 reports only unstandardized coefficients with Wald SEs. With N = 5,114, statistical significance is nearly guaranteed for any nonzero effect. The authors should report standardized effect sizes (partial R², Cohen's f²) for all ADHD associations. Without these, the reader cannot evaluate the clinical relevance of the findings. The abstract and framing should be adjusted to match the "rather weak" associations (line 570).

2. Fixed vs. free parameter choices need justification. Fixing λ = 0.2 (hazard rate) and r̃ = 1/6 (stop prior) while freeing six other parameters is a consequential decision that is inadequately motivated ("based on previous work [21] and pilot testing," line 362). Fig 9 demonstrates that r̃ profoundly affects behavior. Meanwhile, Yu & Dayan (2005; Ref [20]) showed participants adaptively track stop probability, suggesting λ and r̃ could absorb variance currently attributed to other parameters. A sensitivity analysis varying the fixed values, or a formal model comparison, would strengthen the paper.

3. Stop-parameter recovery is moderate — implications for null ADHD findings. Recovery of δ (r = 0.67) and δ' (r = 0.60) is substantially lower than for go-related parameters. The null ADHD association with stop-signal perceptual parameters could therefore reflect estimation noise rather than a true absence of effect. This should be explicitly discussed, as stop-signal sensitivity deficits are a plausible ADHD mechanism (cf. Weigard et al., 2019; Ref [9]).

4. Non-decision time omission confounds parameter interpretation. Without a non-decision time component, the model may attribute motor/encoding latency to Go precision (χ) or inverse temperature (ϕ). This is especially relevant to the ADHD findings, since ADHD has been associated with longer non-decision times in DDM-based analyses of the same ABCD task (Weigard et al., 2023; Ref [42]). This limitation needs more than a one-sentence acknowledgment.

5. No model comparison is provided. The POMDP is positioned as superior to race models for the dependent ABCD design, but this is asserted rather than demonstrated. Even a qualitative PPC comparison against a fitted RDEX-ABCD or DDM model would substantiate the claim.

Minor Points

- Sample size discrepancy: Main text says N = 5,114; Supplementary Table B says N = 5,144. Which is correct?

- Broken reference: Line 633 contains "[?]" — a missing LaTeX citation for time perception noise.

- Wrong symbol (line 429): Text says "stop belief δ^ν_t" but should be "σ^ν_t"? δ is the stop precision parameter.

- Fig 10 caption: "highlighted (teal) model's adaptive behavior" is missing a word.

- Typo (line 310): "modeling the staircase using in ABCD" → "used in ABCD."

- Inconsistent naming: "Section B.2 of Appendix S1" (line 544) vs. "S1 Appendix" elsewhere.

**********

**Have the authors made all data and (if applicable) computational code underlying the findings in their manuscript fully available?**

The PLOS Data policy requires authors to make all data and code underlying the findings described in their manuscript fully available without restriction, with rare exception (please refer to the Data Availability Statement in the manuscript PDF file). The data and code should be provided as part of the manuscript or its supporting information, or deposited to a public repository. For example, in addition to summary statistics, the data points behind means, medians and variance measures should be available. If there are restrictions on publicly sharing data or code —e.g. participant privacy or use of data from a third party—those must be specified.

Reviewer #1: Yes

Reviewer #2: **No:**  the authors said that "all code used for the POMDP modeling, parameter inference, and statistical analyses will be made publicly available on the Open Science Framework" (line 693), but no repository link or deposited code is provided with the current submission.

**********

PLOS authors have the option to publish the peer review history of their article (what does this mean?). If published, this will include your full peer review and any attached files.

If you choose “no”, your identity will remain anonymous but your review may still be made public.

**Do you want your identity to be public for this peer review?**  For information about this choice, including consent withdrawal, please see our Privacy Policy.

Reviewer #1: **Yes:**  Xu Pan

Reviewer #2: No

[NOTE: If reviewer comments were submitted as an attachment file, they will be attached to this email and accessible via the submission site. Please log into your account, locate the manuscript record, and check for the action link "View Attachments". If this link does not appear, there are no attachment files.]

**Figure resubmission:**

After uploading your figures to PLOS’s NAAS tool - https://ngplosjournals.pagemajik.ai/artanalysis, NAAS will process the files provided and display the results in the "Uploaded Files" section of the page as the processing is complete. If the uploaded figures meet our requirements (or NAAS is able to fix the files to meet our requirements), the figure will be marked as "fixed" above. If NAAS is unable to fix the files, a red "failed" label will appear above. When NAAS has confirmed that the figure files meet our requirements, please download the file via the download option, and include these NAAS processed figure files when submitting your revised manuscript.

**Reproducibility:**

To enhance the reproducibility of your results, we recommend that authors of applicable studies deposit laboratory protocols in protocols.io, where a protocol can be assigned its own identifier (DOI) such that it can be cited independently in the future. Additionally, PLOS ONE offers an option to publish peer-reviewed clinical study protocols. Read more information on sharing protocols at https://plos.org/protocols?utm_medium=editorial-email&utm_source=authorletters&utm_campaign=protocols

# Author comment: Author response to Decision Letter 1

**DOI:** 10.1371/journal.pcbi.1014068.r002

14 Jul 2026

Attachment Submitted filename: Response_to_reviewers.pdf

# Peer review: Decision Letter 1

**DOI:** 10.1371/journal.pcbi.1014068.r003

**Authors:** Andrea E. Martin, Jian Liu

10 Sep 2026

Dear Ms. Wang,

We are pleased to inform you that your manuscript 'Decomposing response inhibition: a POMDP model' has been provisionally accepted for publication in PLOS Computational Biology.

Before your manuscript can be formally accepted you will need to complete some formatting changes, which you will receive in a follow up email. A member of our team will be in touch with a set of requests.

Please note that your manuscript will not be scheduled for publication until you have made the required changes, so a swift response is appreciated.

IMPORTANT: The editorial review process is now complete. PLOS will only permit corrections to spelling, formatting or significant scientific errors from this point onwards. Requests for major changes, or any which affect the scientific understanding of your work, will cause delays to the publication date of your manuscript.

Should you, your institution's press office or the journal office choose to press release your paper, you will automatically be opted out of early publication. We ask that you notify us now if you or your institution is planning to press release the article. All press must be co-ordinated with PLOS.

Thank you again for supporting Open Access publishing; we are looking forward to publishing your work in PLOS Computational Biology.

Best regards,

Jian Liu

Academic Editor

PLOS Computational Biology

Andrea E. Martin

Section Editor

PLOS Computational Biology

***********************************************************

The manuscript has been reasonably revised within the current scope of the study and is now suitable for acceptance.

Reviewer's Responses to Questions

**Comments to the Authors:**

**Please note here if the review is uploaded as an attachment.**

Reviewer #1: I would like to thank the authors for responding to my comments. In my previous round of review, my suggestions focused primarily on improving the clarity of the presentation, as I had no major concerns about the scientific value of the manuscript. The revised manuscript is significantly more readable, thanks to the restructured sections, improved schematic diagrams, and plots such as Figure 9, which effectively visualizes the core results. I have no further concerns.

**********

**Have the authors made all data and (if applicable) computational code underlying the findings in their manuscript fully available?**

The PLOS Data policy requires authors to make all data and code underlying the findings described in their manuscript fully available without restriction, with rare exception (please refer to the Data Availability Statement in the manuscript PDF file). The data and code should be provided as part of the manuscript or its supporting information, or deposited to a public repository. For example, in addition to summary statistics, the data points behind means, medians and variance measures should be available. If there are restrictions on publicly sharing data or code —e.g. participant privacy or use of data from a third party—those must be specified.

Reviewer #1: Yes

**********

PLOS authors have the option to publish the peer review history of their article (what does this mean?). If published, this will include your full peer review and any attached files.

If you choose “no”, your identity will remain anonymous but your review may still be made public.

**Do you want your identity to be public for this peer review?**  For information about this choice, including consent withdrawal, please see our Privacy Policy.

Reviewer #1: No

# Editor report: Acceptance letter

**DOI:** 10.1371/journal.pcbi.1014068.r004

**Authors:** Andrea E. Martin, Jian Liu

PCOMPBIOL-D-26-00444R1

Decomposing response inhibition: a POMDP model

Dear Dr Wang,

I am pleased to inform you that your manuscript has been formally accepted for publication in PLOS Computational Biology. Your manuscript is now with our production department and you will be notified of the publication date in due course.

The corresponding author will soon be receiving a typeset proof for review, to ensure errors have not been introduced during production. Please review the PDF proof of your manuscript carefully, as this is the last chance to correct any errors. Please note that major changes, or those which affect the scientific understanding of the work, will likely cause delays to the publication date of your manuscript.

Soon after your final files are uploaded, unless you have opted out, the early version of your manuscript will be published online. The date of the early version will be your article's publication date. The final article will be published to the same URL, and all versions of the paper will be accessible to readers.

For Research, Software, and Methods articles, you will receive an invoice from PLOS for your publication fee after your manuscript has reached the completed accept phase. If you receive an email requesting payment before acceptance or for any other service, this may be a phishing scheme. Learn how to identify phishing emails and protect your accounts at https://explore.plos.org/phishing.

Thank you again for supporting PLOS Computational Biology and open-access publishing. We are looking forward to publishing your work!

With kind regards,

Sharmila Kamatchi

PLOS Computational Biology | Carlyle House, Carlyle Road, Cambridge CB4 3DN | United Kingdom ploscompbiol@plos.org | Phone +44 (0) 1223-442824 | ploscompbiol.org | @PLOSCompBiol
